# Pro-tumorigenic role of lnc-ZNF30-3 as a sponge counteracting miR-145-5p in prostate cancer

**DOI:** 10.1186/s13062-023-00393-7

**Published:** 2023-07-11

**Authors:** Matthieu Le Hars, Luis Jaime Castro-Vega, Fatemeh Rajabi, David Tabatadze, Martha Romero, Marina Pinskaya, Irina Groisman

**Affiliations:** 1 Institut Curie, Sorbonne Universités, Paris Sciences et Lettres Research University, CNRS UMR3244, Paris, France; 2 Paris Brain Institute (ICM), Hôpital Pitié-Salpêtrière, Inserm U1127, CNRS UMR7225, Sorbonne Universités, Paris, France; 3 Cancer Genomics lab, Inserm U981, Gustave Roussy Cancer Center Grand Paris, Villejuif, France; 4 ZATA Pharmaceuticals Inc, Worcester, MA USA; 5 Department of Pathology, Hospital Universitario-Fundación Santa Fe de Bogotá, Bogotá, Colombia

**Keywords:** Prostate cancer, miR-145, lnc-ZNF30-3, EMT, TWIST1

## Abstract

**Background:**

Prostate cancer remains one of the deadliest neoplasms in developed countries. Identification of new molecular markers that predict the onset and progression of the disease could improve its clinical management. Low miR-145-5p expression is consistently found in primary tumors and metastases, but the regulatory mechanisms governing its functions remain largely unknown.

**Methods:**

Bioinformatics analysis was conducted to identify [1] a set of novel potential competing endogenous lncRNAs for sponging of miRNA-145-5p in prostate cancer and [2] miR-145-5p and other EMT-related miRNAs response elements in lnc-ZNF30-3. Quantification of miR-145-5p, lnc-ZNF30-3, and TWIST1 expression levels in tumor tissues in RNA sequencing datasets of our and TCGA PRAD cohorts revealed a correlation with clinical outcome of prostate cancer patients. Biochemical and cell biology approaches, such as RNA pull-down, western blot, immunostaining, and wound healing assays were used for evaluation of the impact of TWIST1/miR-145/ lnc-ZNF30-3 interactions in prostate cancer cells altered in miRNA and lncRNA expression.

**Results:**

We identified a few potential lncRNA sponges of miR-145-5p, including lnc-ZNF30-3. It contains five response elements for miR-145-5p, but also other miRNAs targeting EMT transcription factors. Lnc-ZNF30-3 is significantly upregulated in prostate cancer cell lines and tumor tissues, and its high expression is correlated with poor patient prognosis. We demonstrated that lnc-ZNF30-3 is associated with AGO2 and specifically interacts with the miR-145-5p seed region. Knockdown of lnc-ZNF30-3 results in decreased migration of prostate cancer cells and downregulation of EMT drivers such as TWIST1 and ZEB1 at both the RNA and protein levels. These phenotypic and molecular features of lnc-ZNF30-3-depleted cells are partially rescued by miR-145-5p inhibition.

**Conclusions:**

Collectively, our results point to lnc-ZNF30-3 as a novel competing endogenous lncRNA for miR-145-5p and other miRNAs that target TWIST1 as well as other EMT transcription factors. Prostate cancer patients with high lncRNA expression in primary tumors show lower survival rate suggesting that lnc-ZNF30-3 may contribute to prostate cancer progression and metastasis.

**Supplementary Information:**

The online version contains supplementary material available at 10.1186/s13062-023-00393-7.

## Background

Prostate cancer (PCa) is the second most common cancer in men world-wide and one of the leading causes of cancer-related deaths in developed countries [1]. The androgen receptor is a driver of cellular differentiation and PCa development, and AR signaling is used as a first-line target in PCa treatment by androgen-deprivation therapy. However, in a majority of cases, regardless of chemotherapy and hormonal therapy, PCa progresses to a secondary metastatic and lethal form [2]. The Gleason score, a grading system based on histomorphological criteria, is currently used for predicting PCa prognosis, albeit it is of limited value. Thus, there is an urgent need to identify tumor molecular markers to better stratify patients at risk of progression. Better understanding of the molecular alterations underlying the metastatic phenotype could pave the way to attain this goal.

Activation of the epithelial-to-mesenchymal transition (EMT) program endows epithelial cancer cells with metastatic and chemo-resistant properties. During EMT, the loss of cellular adhesion and remodeling of the extra-cellular matrix, which are important for the maintenance of cell polarity, lead to enhanced cell migration and invasion. These changes in cell properties are orchestrated by core transcription factors: EMT drivers such as TWIST1/2, ZEB1/2, SNAIL1/2, TGF-β1, and FOXC2 [3]. A basic helix-loop-helix transcription factor, TWIST1, is an important regulator of embryogenesis [4–6], whereas in adult tissues its expression is restricted to mesenchymal stem cells [6]. Increased TWIST1 expression has been detected in several cancers, including PCa [6, 7], where it promotes escape from senescence [8], tumor initiation, metastasis, stemness, and drug resistance [5]. Thus, TWIST1 seems to orchestrate transcriptional programs of both mesenchymal and epithelial genes that drive EMT reprogramming of cancer cells. It has been shown that TWIST1 expression can be regulated by miRNAs such as miR-145-5p, targeting its 3’UTR [42,41,40]. This miRNA was proposed as a tumor suppressor gene whose downregulation in numerous cancers including PCa [9–11] promotes proliferation, angiogenesis, and metastasis [12–15]. Mechanistically, downregulation of miR-145-5p in epithelial cells can lead to increased TWIST1 expression and subsequent activation of the EMT program.

Accumulating evidence indicates that lncRNAs can regulate miRNA functions. We and others have reported that lncRNAs are aberrantly expressed in PCa and might contribute to tumor initiation and progression [16–20]. One of the documented functions of lncRNAs is to act as miRNA sponges or competing endogenous (ce)RNAs that decoy miRNAs from mRNA targets [17, 21]. CeRNAs usually contain several miRNA response elements (REs) binding several miRNA molecules simultaneously and changing their balance in cells and consequently expression of their protein-coding gene targets. In cancer, crosstalk between lncRNAs, mRNAs, and miRNAs constitutes a fine-tuning regulatory circuit that controls gene expression and drives tumor growth. Importantly, this operational mode has been proposed for miR-145 and several different lncRNAs in various cancers. In PCa, only PCGEM1 has been experimentally demonstrated to be a sponge for miR-145 [22].

Herein, using computational predictions and transcriptomic analysis of tumor tissues, we identified a set of lncRNAs that overexpress in PCa and contain response elements (REs) for miR-145-5p. *In vitro* pull-downs with biotinylated miR-145-5p confirmed miR-145 binding to several lncRNAs in PCa cells lysates. Among those, lnc-ZNF30-3, annotated in the GENCODE database but not yet described in the literature, was the most enriched lncRNA. Analysis of the PRAD cohort of The Cancer Genome Atlas data confirmed that low levels of lnc-ZNF30-3 correlate with progression-free survival in PCa patients. We showed that expression of this lncRNA is associated with increased levels of TWIST1 in PCa cells. Further bioinformatics analysis revealed that in addition to miR-145-5p, lnc-ZNF30-3 contains REs for other miRNAs experimentally proven to target TWIST1 in various cancers and cell models. Moreover, lncZNF30-3 matches seed regions of several miRNAs targeting TWIST2, SNAIL1/2, and ZEB1/2 EMT transcription factors. Accordingly, knockdown of lnc-ZNF30-3 reduces the expression of EMT drivers TWIST1 and ZEB1 and decreases migration of PCa cells. Altogether, our findings indicate that high expression of lncRNA lnc-ZNF30-3 contributes to the activation of the EMT program and to a poor clinical outcome in PCa patients by counteracting the tumor suppressive role of miR-145-5p and other EMT-related miRNAs.

## Methods

### Cell culture

All prostate cell lines: PC3a, PNT1A, 22Rv1, and LNCaP, DU145 were obtained from ATCC and cultured in RPMI media (ThermoFisher) with 10% FBS.

### FFPE cohort of PCa tissues

Formalin-fixed paraffin-embedded (FFPE) tissues from 16 patients with newly diagnosed PCa, treated by radical prostatectomy and pelvic lymph node dissection (Supplementary Table 1), and five normal prostates from necropsies of age-matched individuals, were included in this study. Informed consent was obtained in accordance with the Declaration of Helsinki. This study was approved by the Fundación Santa Fe Institutional Review Board. The median age at the time of diagnosis was 57.5 ± 8.2 (range 42–70) years. Patients were stratified into risk groups according to D’Amico Classification [23]. Four patients were low risk; four patients were intermediate risk; and eight patients were high risk, including four who developed lymph node metastases). Histological diagnosis and Gleason scores were recorded according to the consensus criteria [24]. Manual microdissection of all FFPE samples was carried out in order to obtain a pure population of epithelial cells for miRNA expression analysis.

### Pathological analyses

Using a microtome, FFPE tissue sections were cut in 5 μm-thick slices and put on electrostatically-charged microslides. Hematoxylin and eosin staining were carried out using standard procedures to examine tissue morphology for diagnosis and to perform microdissection of the epithelial compartment for miRNA expression analysis. In short, tissue sections were deparaffinized in xylene, hydrated gradually in a series of graded alcohols, and washed in deionized water. Slides were stained with Gill’s hematoxylin solution, then washed in acid alcohol and rinsed with water overnight, after which eosin staining and dehydration were carried out. Immunohistochemistry for E-cadherin, Vimentin, and Ki-67 was done on deparaffinized sections, using an indirect immunoperoxidase method and Dako antibodies against these markers. In short, slides were treated with a sodium citrate buffer for antigen unmasking at pH 6.0, heated at 95°C for 5 min, and washed in deionized water. Slides were incubated with HRP-conjugated IgG followed by incubation in a peroxidase substrate and chromogen mixture. Optimal time for staining was determined for each antibody. Images were taken using the Provis-AX-70 microscope at 400x magnification with a 0.344 mm field size.

### Transfection of siRNA, miR mimics and inhibitors

A total of 30 pmol of siRNA, of 30 pmol, or 100 pmol of single- or double-stranded biotinylated probes, respectively, were transfected into 3*10^5^ PC3a cells or 6*10^5^ PNT1A cells, using RNAi RNAmax reagent (Qiagen) according to the manufacturer’s instructions. Experiments were carried out 24 h post-transfection.

MiR-145-5p mimic (MC11480), inhibitor (MH11480) and mi-scr (AM17010) were purchased from Ambion; Lnc-ZNF30-3 siRNA 5’ ugcaagcaguccagguguauu 3’ (HR1ZN-004520) from Dharmacon; scrambled siRNA (siRNAscr-1 529,856) from Sigma.

The 2’F modified mimics, named ZATA-miRs, were provided by ZATA Pharmaceuticals Inc. Mimics were biotinylated using the 3’end Biotinylation Kit (PierceTM) according to the manufacturer’s instructions. Biotinylated mimics were annealed with the equimolar amount of a complementary passenger strand by heating to 70°C for 3 min and progressive cooling down to 21°C.

### Wound healing assay

The scratch was introduced into a confluent monolayer of cells with a pipette tip and cell migration was measured 24 h post-scratch. Images were quantified using TScratch software as a wound surface at 24 h (T_24_) versus at time zero (T_0_) post-scratch.

### Western blot

Cells were detached from plates by trypsinization, pelleted, and washed once in phosphate-buffered saline (1xPBS, Life Technologies), then resuspended in the lysis buffer (20 mM Hepes-KOH pH 7.8, 100 mM KCl, 5 mM MgCl2, 2 mM DTT, 25% NP-40) supplemented with a protease inhibitor cocktail (Roche) and incubated on ice for 15 min. The lysate was centrifuged at 13,000 g for 30 min and the supernatant was recovered. A total of 20 µg of proteins was resolved in Bis-Tris polyacrylamide gel 4–12% (Nu-Page, Invitrogen) and transferred to a nitrocellulose membrane (Amersham) using the Xcell II blot module (Invitrogen). Membranes were stained using Ponceau Red (Sigma) then washed in PBST (1xPBS, 0.1% Tween-20, Sigma) and blocked in PBST with 5% non-fat milk. Primary antibodies (dilution, reference, supplier): Vimentin (1:1000, 9782, Cell Signaling), E-Cadherin (1:1000, 9782, Cell Signaling), TWIST1 (1:1000, sc-15,393, Santa Cruz Biotechnology), ZEB1 (1:1000, TA802298, Origene), AGO2 (1:1000, Sab42000085V, Sigma), β-Actin (1:2000, sc-4778, Santa Cruz Biotechnology) were incubated overnight at 4 °C in PBST with 5% non-fat milk. Then membranes were washed 3 times for 10 min in PBST and incubated with secondary antibodies in PBST with 5% non-fat milk for 1 h at room temperature. Signals were revealed using the Chemiluminescence kit (SuperSignal, West Femto, Thermo Scientific) and the ChemiDoc Imaging system (Bio-Rad), and quantified using ImageJ software.

### Dot blot

Samples were spotted onto a nylon membrane (GE Healthcare) and air-dried. The membrane was then baked in a regular oven at 80°C for 2 h and exposed to ultraviolet radiation for 2 h. The membrane was incubated in blocking solution with 5% milk in PBST for 1 h at room temperature. It was then incubated with HRP-conjugated streptavidin (TSATM KIT, Invitrogen) overnight in 5% milk in PBST and washed 3 times in PBST buffer. The dot blot was developed using a chemiluminescence kit (SuperSignal, West Femto, Thermo Scientific) and the ChemiDoc imaging system (BioRad).

### RNA extraction, reverse transcription, and quantitative *PCR (RT-qPCR)*

Total RNA was extracted from cells using Qiazol (Qiagen) according to the manufacturer’s instructions. Either 1000 ng of total RNA or 200 ng of DNase-treated total RNA was used for random primed transcription with SuperScript™ III Reverse transcriptase (Thermo Fisher). cDNAs were quantified by qPCR using the SYBR Green GoTaq^R^ Master Mix (Promega) and the Roche LC480 instrument.

From FFPE samples (20-µm-thick paraffin sections), total RNA was extracted using the Recover All total nucleic acid isolation kit (Ambion). MicroRNA expression was examined via RT-qPCR following the MIQE guidelines [25] and Taqman assays targeting hsa-miR-145-5p and RNU24 as a reference gene (Applied Biosystems). Data were obtained using the LightCycler® 480 Real-time PCR System (Roche Applied Science). Only CT values below 35 were considered for analysis. Fold-change was calculated using the ${2}^{-{\Delta }{\Delta }\text{C}\text{T}}$ method relative to ACTB/G1 for lncRNAs and mRNAs and RNU24 for miRNAs [26]. Primer sequences are presented in Supplementary Table 2.

### RNA pull-down

Cell pellets were incubated with the RIPA buffer (Pierce™) supplemented with the protease inhibitor cocktail (Roche) and Superase In, RNase inhibitor (Thermo Fisher Scientific) for 15 min on ice, then centrifuged for 15 min at 13,000 rpm. Supernatants were collected and supplemented by an equal volume of 2X TNT buffer (50 mM Tris-Cl pH 8.0, 2 mM EDTA, 150 mM NaCl, and 1% Triton X-100). Streptavidin agarose beads (30 µl per reaction, Thermo Fisher Scientific) were washed in TNT 3 times for 2 min and 2,000 rpm spin at 4ºC, then incubated with cellular extracts for 1 h at room temperature. Beads were washed 3 times for 10 min at 20 rpm in 500 µL PBS and treated with Qiazol for RNA extraction.

### Bioinformatics search for miR-145 RE in lncRNAs

The transcripts were searched for canonical (6mer, UCCAGU) miR-145-5p seed motif matches RE (ACUGGA) (Friedman et al., 2009). Target search and result visualization were carried out using an *in-house* developed tool in R (R Core Team (2013).

### RNA-sequencing datasets

The PAIR cohort data for lncRNAs is composed of 24 RNA-seq datasets (16 tumor and 8 normal prostate tissues of prostatectomy origin) and were retrieved from the gene omnibus portal, accession number GSE115414. RNA-seq data and corresponding clinical information were obtained from a publicly available TCGA dataset of the PRAD cohort comprised in total of 424 specimens (52 normal and 372 tumor tissues) (http://cancergenome.nih.gov).

## Results

### MiR-145-5p is downregulated in advanced and poor prognosis prostate cancer

**Fig. 1:**
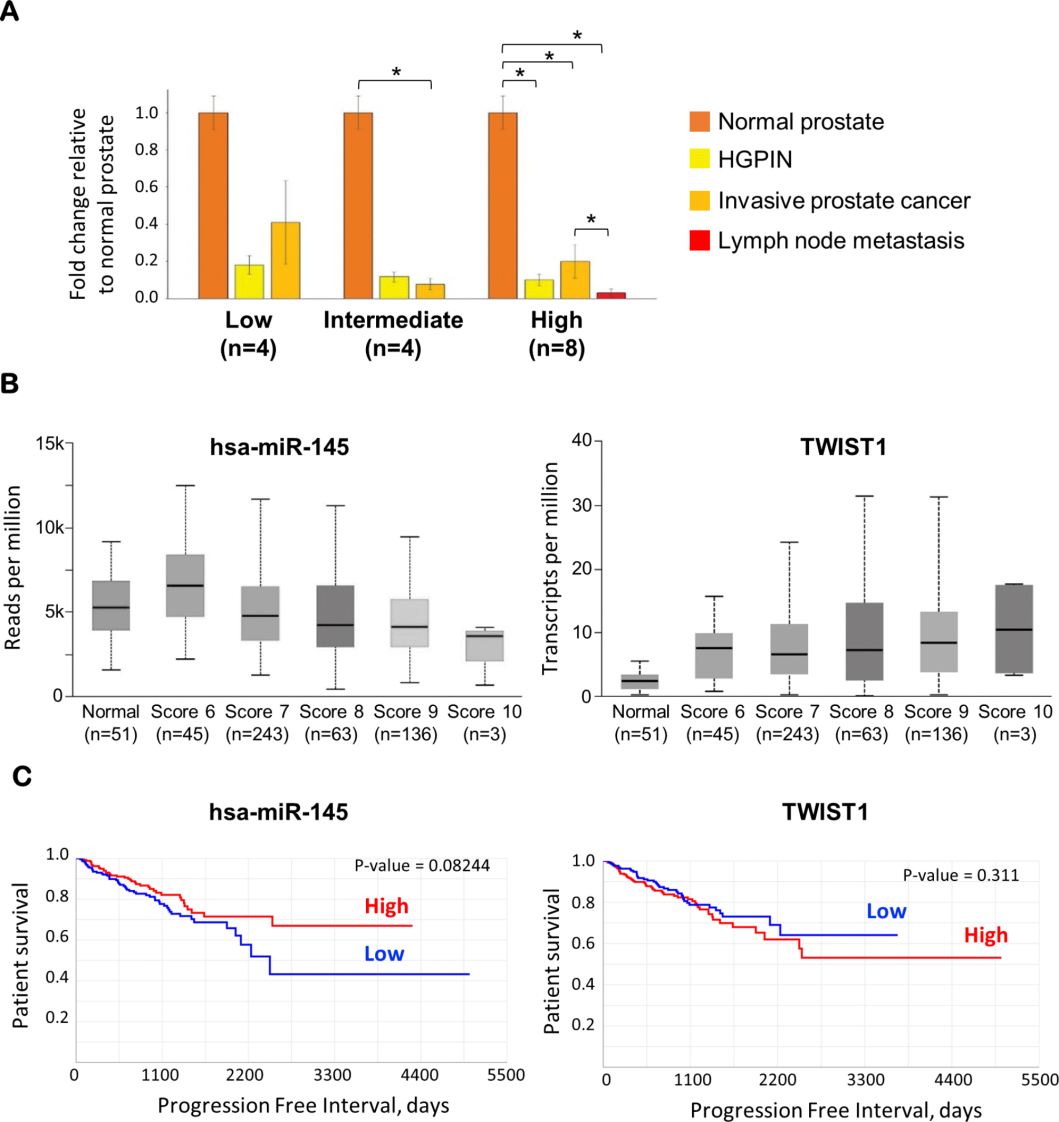
Expression patterns of miR-145-5p, TWIST1 and EMT markers is associated with different stages of prostate cancer progression and prognosis. **A.** Expression of miR-145-5p in the epithelial compartment of PCa tissues annotated as Gleason Low (n = 4), Gleason Intermediate (n = 4) and Gleason High (n = 8)-grade, relative to normal prostate (n = 5). Four patients with Gleason High-grade pathology developed metastases. * P-value < 0.05 (Kruskal-Wallis test; error bars indicate mean +/- sem). **B**. Quantification of miR-145-5p and TWIST1 mRNA levels in the TCGA PRAD data according to the Gleason score [28]. **C**. Survival analysis of the TCGA PRAD cohort based on expression data of miR-145-5p and TWIST1.

Analysis of microdissected FFPE PCa tissues from 16 patients and five normal prostates used as controls revealed significant downregulation in the expression of miR-145-5p in high-grade prostatic intraepithelial neoplasia (HGPIN) and invasive PCa, histologically classified as tumors of Gleason grade High (Fig. 1A, Supplementary Table 1). Notably, this downregulation was more pronounced in lymph node metastases. MiR-145-5p expression pattern correlated with the activation of the EMT program, as indicated by concomitant upregulation of the mesenchymal marker Vimentin and the proliferation marker Ki-67, and downregulation of the epithelial marker E-Cadherin (Supplementary Fig. 1A). Analysis of the TCGA PRAD cohort confirmed that expression of miR-145-5p is progressively downregulated during tumor progression, particularly at advanced stages, and correlates with poor prognosis (Fig. 1B and C). Of note, this change in the miR-145-5p levels is not explained by copy number loss, since this locus instead appears to be amplified in up to 3% of cases across 10 various PCa cohorts of cBioPortal [27] (Supplementary Fig. 1B). Conversely, expression of TWIST1, a well-known target of miR-145-5p, is upregulated during PCa progression, but has limited prognostic value (Fig. 1B and C). High expression of TWIST1 can be partially explained by amplification in up to 10% of PCa cases of cBioPortal (Supplementary Fig. 1B). Collectively, these observations suggest that additional regulatory mechanisms of the miR-145 and TWIST1 axis might be at play during PCa development. Among others, we hypothesized that lncRNAs can counteract the action of miR-145-5p in a control of TWIST1 levels.

### Several lncRNAs contain response elements and bind miR-145-5p in PCa cells

**Fig. 2:**
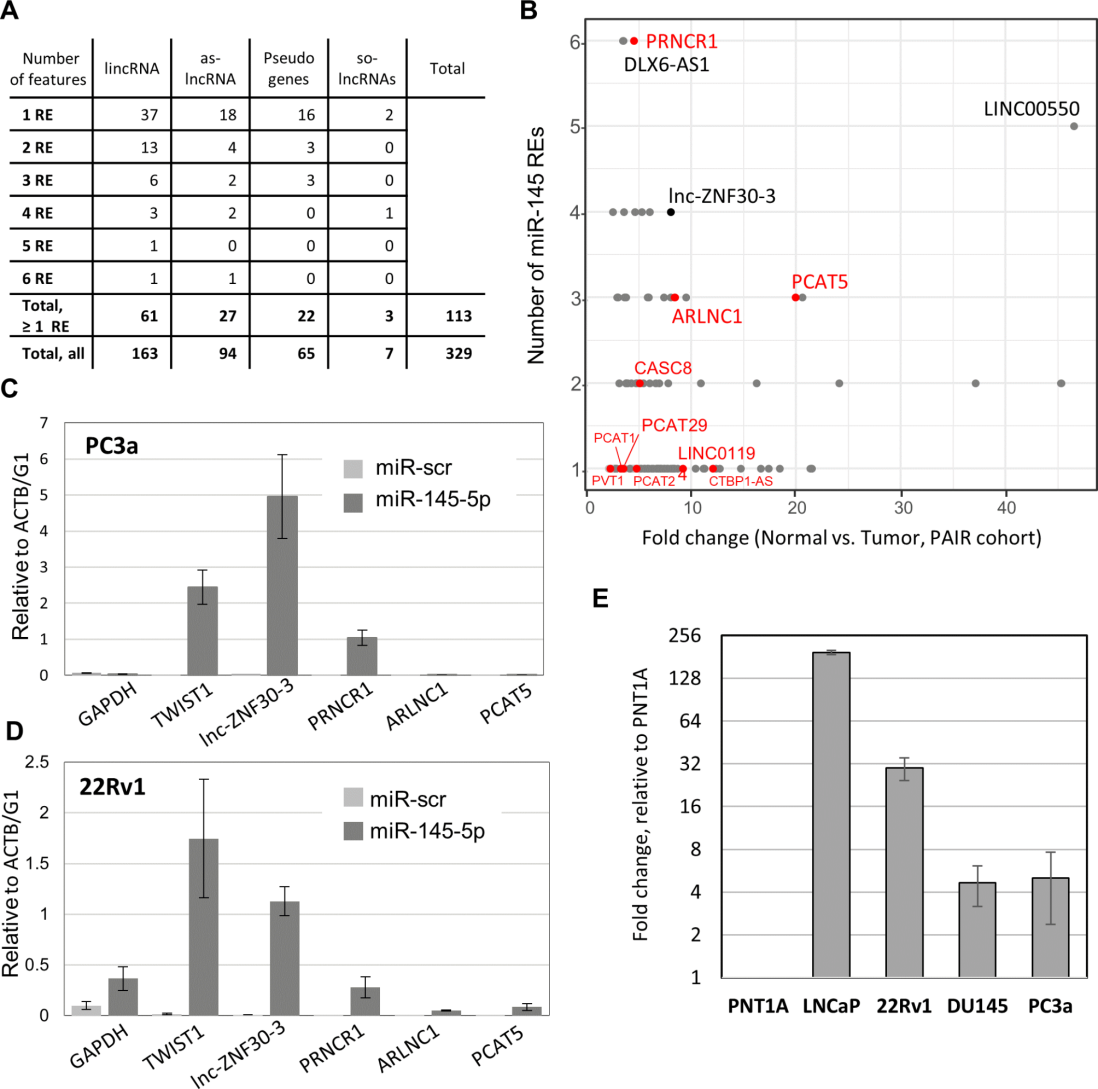
Identification of PCa-associated lncRNAs containing REs and interacting with miR-145 in PCa cells. **A.** Summary table representing different types of PCa-associated lncRNAs and a number of RE for the canonical miR-145-5p seed motif (UCCAGU) identified by bioinformatics analysis of seed match. As-lncRNA – antisense lncRNA, so-lncRNA – sense overlapping lncRNA. **B. **2D plot of miR-145-5p conventional RE sequence occurrence and the respective fold- change of lncRNA expression in prostate cancer (n = 16) compared with normal tissues (n = 8). LncRNA annotation includes long intergenic noncoding (linc)RNAs and antisense lncRNAs (GENCODE v26). LncRNAs associated with PCa in previous studies are labeled in red; lnc-ZNF30-3 is labeled in black. **C-D.** RT-qPCR quantification of lncRNAs co-precipitated with biotin-labeled miR-145 mimics on streptavidin beads from PC3a (**C**) and 22Rv1 (**D**) cells. **E**. Enrichment of lnc-ZNF30-3 in PCa cell lines LNCaP, 22Rv1, DU145, PC3a, and immortalized normal prostate cell line PNT1A normalized by PNT1A presented in log2 scale.

To find lncRNAs with a capacity to bind miR-145-5p in PCa tissues we carried out gene expression and miRNA seed-match analyses, using our previously published total RNA-sequencing dataset from 16 tumor and 8 contralateral normal prostate tissues [16]. First, we isolated 329 lncRNAs that showed higher expression in tumors compared with normal tissues (fold change ≥ 2, and p-value ≤ 0.01) (Supplementary Table 3). Among them we found such lncRNAs as PCAT1 and PCAT2, already identified in the ceRNA network [29], but the majority were transcripts with yet unknown roles in PCa biology. We hypothesized that some of these RNAs may interfere with miR-145-5p functions through ceRNA activity, ultimately contributing to PCa progression and aggressiveness. To test this hypothesis, we conducted bioinformatics analysis to isolate 114 transcripts containing at least one miR-145-5p RE from the defined set of 329 PCa-associated lncRNAs (Fig. 2A, Supplementary Table 3). Among them, long intergenic (lincRNAs) and antisense lncRNAs were the most prevalent among various lncRNA types possessing more than one RE (Fig. 2B). Following manual curation of RNA-sequencing reads distribution, genomic position, strength and fold-change of expression between tumor and normal tissues, we selected several lncRNAs for further study. Among the selected ceRNA candidates were PRNCR1, PCAT5, and LINC02170/ARNCR1, well-known PCa-associated lncRNAs [30–32], but also several lncRNAs of yet unknown function (Supplementary Table 3).

To verify our predictions, we tested whether a biotinylated miR-145-5p mimic is able to pull-down ceRNA candidates from cell extracts. Two prostate cancer cell lines, PC3a and 22Rv1, were transfected with *in vitro* biotinylated miR-145 or a control scramble miRNA, miR-scr, and precipitated on streptavidin beads. In PC3a cells, this experiment revealed miR-145 association with the TWIST1 mRNA, an experimentally proven miRNA target, but not with ACTB/G1 or GAPDH mRNAs, used as negative controls. Importantly, three lncRNAs, PRNCR1 (6 REs), DLX6-AS1(6 REs), and lnc-ZNF30-3 (5 REs), were significantly enriched in miR-145 pull-downs, as though PCAT5 (3 REs) and ARNCR1 (3 REs) were barely detectable (Fig. 2C). Comparable results were obtained in the second 22Rv1 cell line (Fig. 2D). Lnc-ZNF30-3 showed the strongest enrichment in miR-145 pull-downs. This lncRNA was found to be expressed in all tested PCa cell lines: PC3a, 22Rv1, DU145, and LNCaP, at higher levels than in the PNT1A cell line immortalized from normal prostate epithelium (Fig. 2E). Hence, we concluded that among tested lncRNAs lnc-ZNF30-3, containing several miR-145 REs, indeed interacts with miR-145 in PCa cells.

### Lnc-ZNF30-3 can potentially sponge miR-145, but also other EMT-related miRNAs, contributing to poor prostate cancer clinical outcomes

**Fig. 3:**
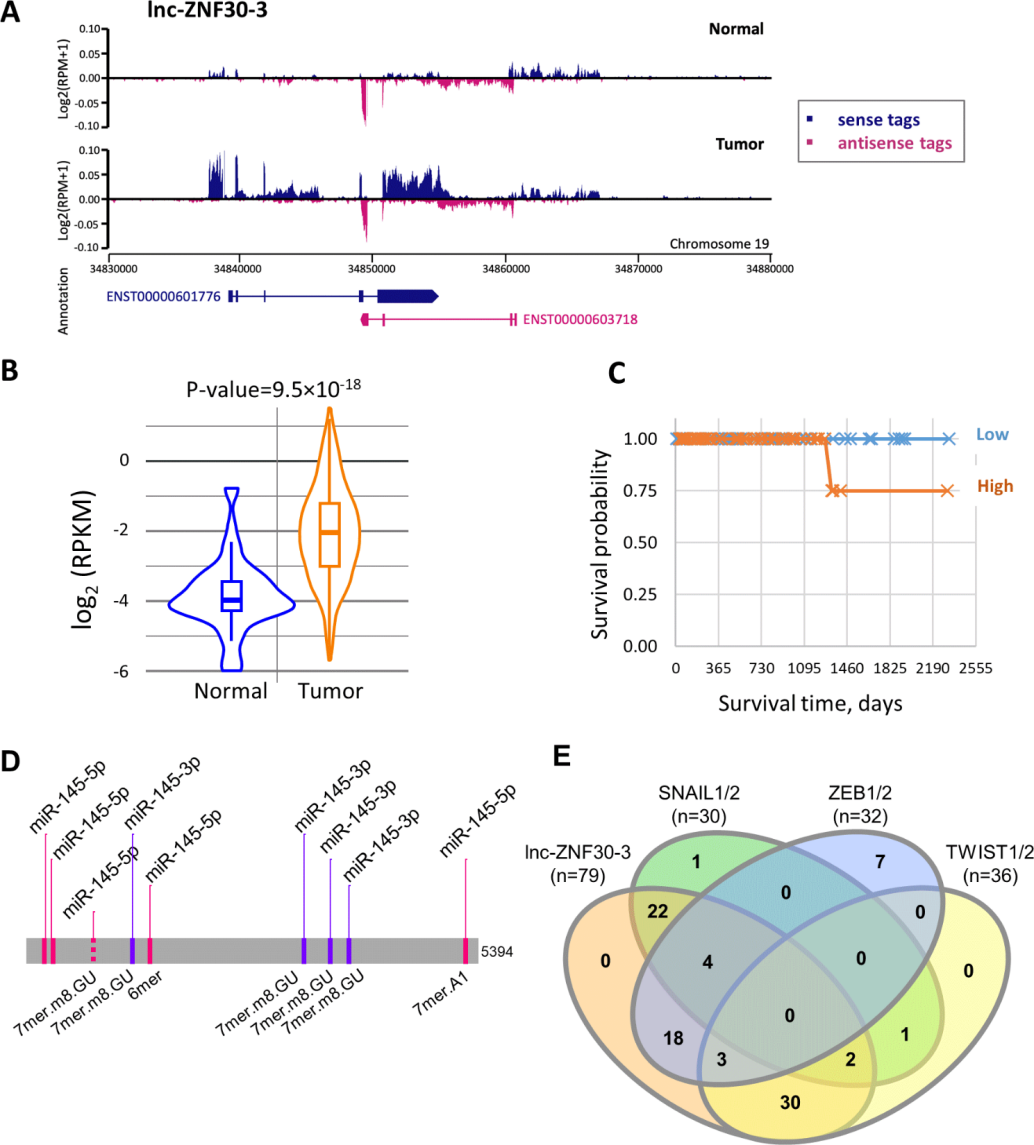
Lnc-ZNF30-3 is upregulated in PCa tumor tissues and potentially correlates with poor-prognosis PCa patients. **A**. RNA-seq profiling of lnc-ZNF30-3 in normal and tumor tissues along sense (blue) and antisense (pink) strands of chromosome 19. **B**. Violin diagram of log2(RPKM) counts of lnc-ZNF30-3 expression in prostate cancer tumor (orange) and normal (blue) tissues of the TCGA-PRAD cohort. Horizontal bars correspond to a mean value. P-value (9.5 × 10^− 18^) was calculated using a Wilcoxon test. **C**. Kaplan-Meier estimation of the PCa patients’ survival related to lnc-ZNF30-3 expression, done using the TANRIC platform and TCGA datasets (P-value 0.11385). **D.** Mapping of potential miR-145 REs along the longest lnc-ZNF30-3 isoform of 5,394 nucleotides. MiR-145-5p 6mer refers to conventional RE sequence (ACUGGA), 7mer.A1 refers to ACUGGAA, 7mer.m8.GU refers to GACUGGA. MiR-145-3p 7mer.m8 refers to GGGAAUC. **E.** Venn diagram representing miRNAs experimentally shown to target TWIST1/2, ZEB1/2, SNAIL1/2 transcription factors, and putative RE within lnc-ZNF30-3.

Lnc-ZNF30-3 is annotated as a long intergenic noncoding RNA gene located in chromosome 19 (ENSG00000269086). Several isoforms are annotated and meet the transcript support level tag in Ensemble, including the one of 5,394 nucleotides (ENST00000601776.2, AC008555.2) hereafter named lnc-ZNF30-3. The latter transcript showed the best RNA-sequencing reads density distribution in prostate tumor tissues of the PAIR cohort (Fig. 3A). It was significantly over-expressed in PCa tumors of the PAIR [16] (Fig. 3A), and the TCGA PRAD cohorts (Fig. 3B). Moreover, high levels of lnc-ZNF30-3 expression correlated with poor clinical outcome in advanced PCa patients of the PRAD cohort (Fig. 3C). Hence, miR-145 and lnc-ZNR30-3 collectively, show negative correlation of expression in advanced PCa tumors, reinforcing the idea of the lncRNA function as a sponge.

More detailed bioinformatics analysis for miR-145-5p seed matching within the lnc-ZNF30-3 transcript identified one supplementary RE, a 7-mer sequence with an additional GU base pair, alone with four conventional 6-mers, but also four miR-145-3p 7-mer GU REs [33] (Fig. 3D, Supplementary Fig. 2). In addition to miR-145, TWIST1 has been experimentally proven to be targeted by 35 other miRNAs in various cancers and cell models (Supplementary Tables 4, Supplementary Fig. 2). Hence, we extended our seed match search and found REs for 34 additional miRNAs within the lnc-ZNF30-3 transcript (Fig. 3E, Supplementary Table 4, Supplementary Fig. 3). In addition to TWIST1 targeting miRNAs, we also curated other miRNAs experimentally proven to control the expression of TWIST2, ZEB1/2, and SNAIL1/2 transcription factors. The majority of seed-matched sites were shared with lnc-ZNF30-3 (Fig. 3E, Supplementary Table 5). The presence and versatility of such an important number of REs within lnc-ZNF30-3 suggests a complex regulatory network between this lncRNA, miRNAs and transcription factors driving EMT.

### Lnc-ZNF30-3 binding to miR-145-5p depends on its seed element

**Fig. 4:**
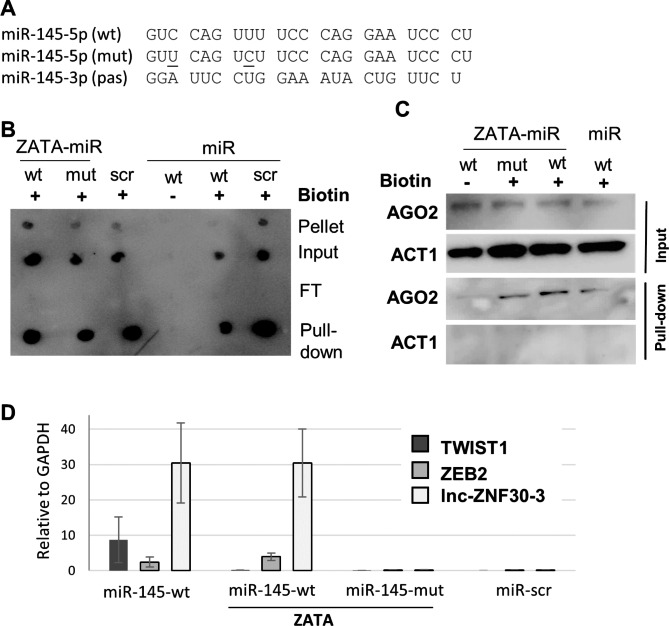
MiR-145-5p specifically binds to lnc-ZNF30-3 in PC3a cells. **A.** Sequences of miRNA mimics (5’ -3’): wt – wild-type consensus of miR-145-5p, mut – mutated sequence containing two nucleotide substitutions (underlined), pas – passenger miR-145-3p strand. **B**. Dot blot analysis of biotinylated (+) and non-biotinylated (-) miR-145-5p detected in PC3a lysates 24 h post-transfection in pellet (cell debris), input (total cell lysate), FT (flow through) and pull-down (streptavidin beads). **C**. Western blot analysis of AGO2 precipitated by biotinylated (+) or non-biotinylated (-) miR-145-5p primed streptavidin beads; ACT1 was used as a negative control. **D**. RT-qPCR quantification of TWIST1 and ZEB2 mRNAs, and lnc-ZNF30-3 co-precipitated by streptavidin beads, primed with biotinylated miR-145-5p wt, mut, and scr sequences.

To further prove the specificity of miR-145-5p and lnc-ZNF30-3 interactions, we carried out a pull-down assay, using biotinylated wild-type (wt) or mutant (mut) miR-145-5p sequence with two nucleotide substitutions in the seed element. In addition, these synthetic miRNAs, hereafter named ZATA-miRs, also contained a 2’F modification of the sugar backbone to improve miRNA penetration into cells (Fig. 4A). A biotinylated synthetic miR-145-5p oligonucleotide and a scramble (miR-scr) were used as controls. Each of the three mimics was annealed to a corresponding non-labeled passenger strain (pas), miR-145-3p (Fig. 4A). We then transfected ZATA and unmodified miR-145-wt and -mut mimics into PC3a cells and carried out streptavidin-mediated precipitations. To verify the efficiency of transfection and precipitation, we transferred an aliquot of each cell lysate collected 24 h post-transfection to a nitrocellulose membrane for blotting with HRP-conjugated streptavidin. Most of the biotinylated miR-145-wt and -mut mimics were detected in cell lysates and highly enriched in streptavidin pull-downs (Fig. 4B). Next, we verified whether the labeled mimics were incorporated into the RISC complex. We carried out Western blot analysis of miR-145-5p-primed streptavidin pull-downs with anti-AGO2 antibody and found that only the biotin-labeled miR-145-5p, ZATA, or unmodified, co-precipitated with AGO2, but not the non-biotinylated one. No ACT1/Actin co-precipitation with biotinylated mimics was detected (Fig. 4C). These experiments demonstrated that biotin-labeled mimics penetrated into cells and were successfully incorporated into the RISC complex. RT-qPCR quantifications revealed the presence of lnc-ZNF30-3 as well as TWIST1 and ZEB2 (proven miR-145 targets) in both miR-145-wt pull-downs, but not in the miR-145-mut mimics and miR-scr controls (Fig. 4D).

This result confirmed a high specificity of interaction between miR-145-5p and lnc-ZNF30-3 requiring the match between miRNA seed and lncRNA RE sequences. Hence, we showed that in PCa cells the lnc-ZNF30-3 interaction with miR-145-5p requires miRNA seed sequence.

### Lnc-ZNF30-3 controls TWIST1 expression and mesenchymal traits of PC3a cells

**Fig. 5:**
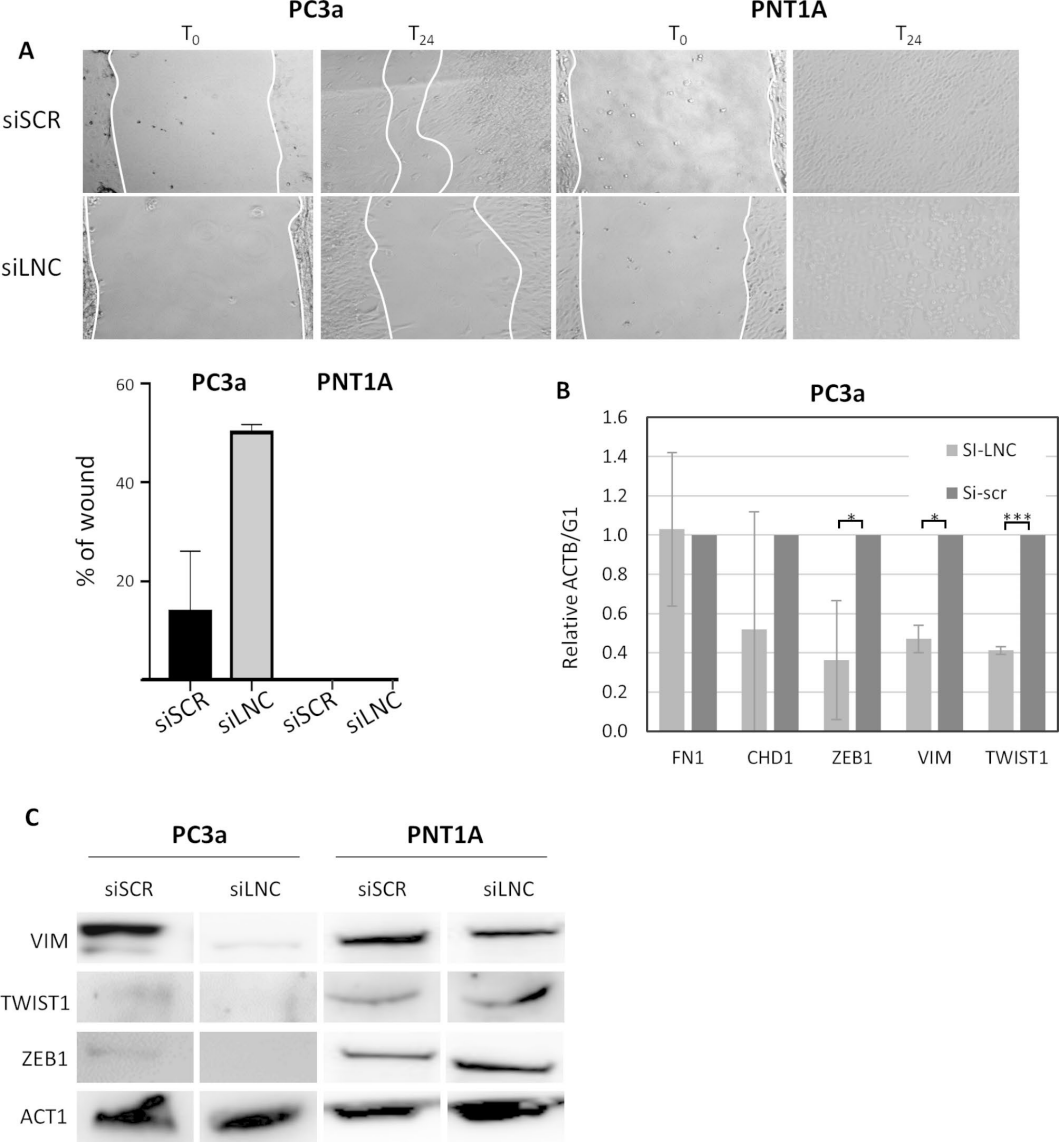
Lnc-ZNF30-3 promotes EMT in PC3a but not in PNT1A cells. **A**. Representative images of cell monolayers at time zero (T_0_) and 24 h post-scratch (T_24_) and quantification of the percentage of an unhealed wound at T_24_ in PC3a and PNT1A cells, transfected with siSCR or siLNC. White lines delineate wound edges. **B.** RT-qPCR quantification of EMT markers expression in PC3a cells transfected with siSCR or siLNC. Each bar corresponds to a mean ± SD relative to ACT1 expression for 3 replicates. *** and * correspond to p-values below 0.001 and 0.05, respectively. **C.** Western blot analysis of EMT markers expression in PC3a and PNT1A cells transfected with siSCR or siLNC.

To understand whether lnc-ZNF30-3 has a function in PCa, we used an siRNA-mediated loss-of-function approach. PC3a or PNT1A cells were transfected with siRNA against lnc-ZNF30-3 (siLNC) or control siRNA (siSCR) and tested for cell migration in a wound-healing assay for further 48 h. PNT1A cells, which do not express lnc-ZNF30-3, were used as a negative control. Depletion of lnc-ZNF30-3 resulted in decreased migration of PC3a cells, whereas it did not have a significant impact on the wound healing of PNT1A cells (Fig. 5A). Loss of cell motility can result from a change in EMT gene expression. We measured the expression of a set of mesenchymal and epithelial markers at the RNA level by RT-qPCR. Depletion of lnc-ZNF30-3 induced a decrease in mRNA levels of mesenchymal Vimentin (VIM), TWIST1 and ZEB1, but not of Fibronectin (FN1) and epithelial E-Cadherin (CHD1) (Fig. 5B). We also measured changes in protein levels of some EMT markers following lnc-ZNF30-3 depletion. Expression of ZEB1, TWIST1, and Vimentin were decreased in PC3a cells but not in PNT1A cells (Fig. 5C). The reduced expression of these mesenchymal genes was consistent with a loss of migration by PC3a depleted of lnc-ZNF30-3, supporting a specific role of this lncRNA in EMT. Notably, it resulted in a partial change of cell identity toward a more pronounced epithelial phenotype.

**Fig. 6:**
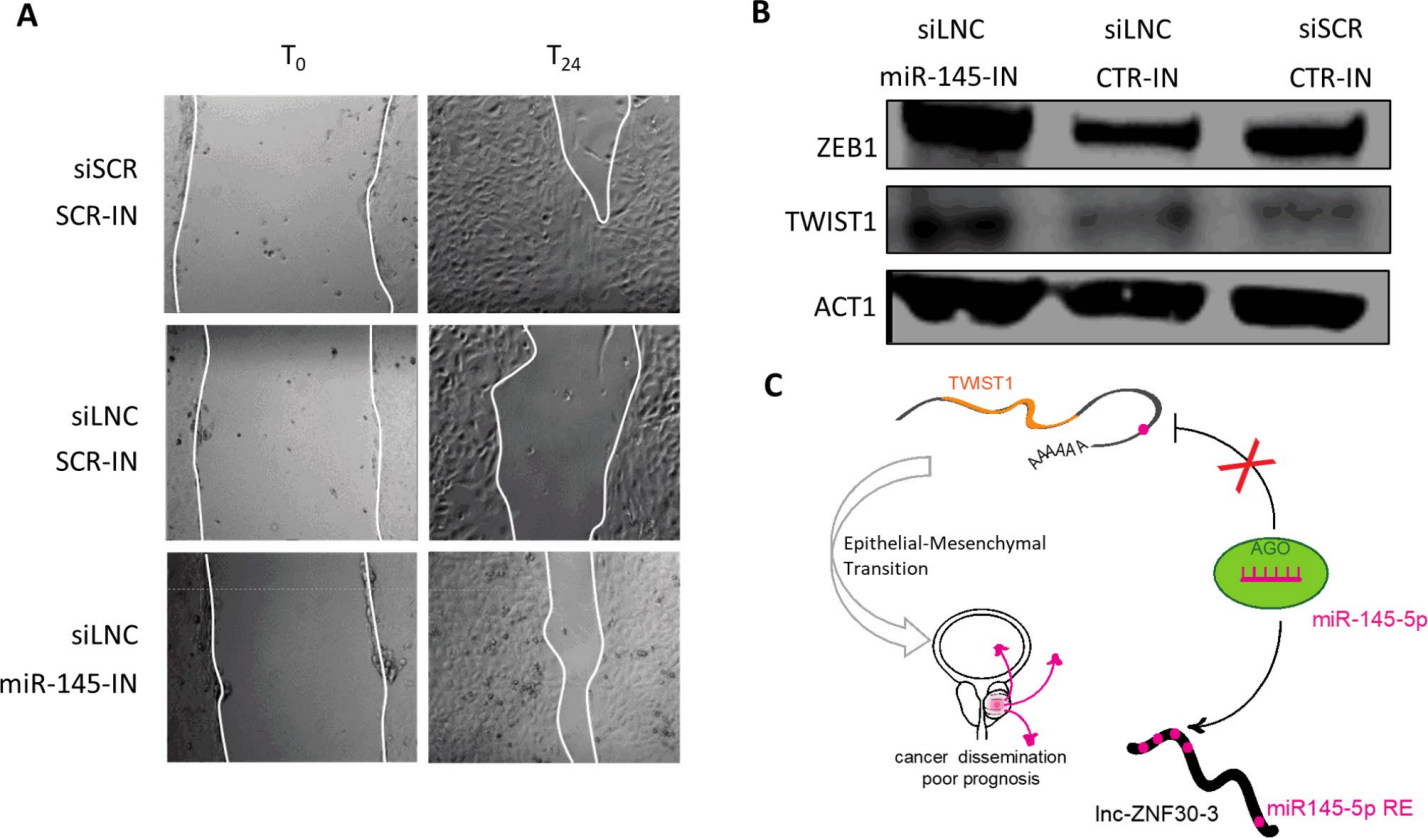
Inhibition of miR-145-5p in lnc-ZNF30-3 depleted PC3a cells rescues cell migration and TWIST1 expression levels. **A.** Images of cell monolayers and quantification of wound healing in PC3a cells 24 h post-scratch transfected with siSCR or siLNC together with control (CTR-IN) or miR-145 inhibitor (miR-145-IN). **B.** Western blot and quantification of TWIST1 and ZEB1 protein levels in PC3a cells transfected with siSCR or siLNC together with scramble (CTR-IN) or miR-145 inhibitors (miR-145-IN). **C.** Model for miR-145-5p, lnc-ZNF30-3 and TWIST1 crosstalk in regulation of EMT properties of PC3a cells that can contribute to cancer progression and dissemination.

We tested whether lnc-ZNF30-3 depletion releases miR-145-5p, which in turn would be able to target and diminish TWIST1 levels, affecting cell motility. PC3a cells were transfected with lnc-ZNF30-3 siRNA alone or together with miR-145-5p inhibitors and used for protein extraction and western blot and, in parallel, for the wound healing assay. We observed that miR-145-5p inhibition resulted in a partial rescue of downregulation of TWIST1 and ZEB1, and consequently of cell motility upon lnc-ZNF30-3 depletion (Fig. 6A and B).

Together, our results supported a role for lnc-ZNF30-3 as the ceRNA diverting miR-145-5p from its genuine target, TWIST1, in a control of EMT in PCa cells (Fig. 6C).

## Discussion

Several studies have shown aberrant expression of miRNAs in PCa, including downregulation of miR-145 [34]. It has been proposed to exhibit tumor suppressive functions by targeting a cell adhesion protein CDH2 [35] or a proto-oncogene WIP1[36]. In the present work, we have confirmed the association of miR-145-5p downregulation with increasing Gleason grade and poor outcome in PCa patients, and identified a novel long noncoding RNA lnc-ZNF30-3 that has ceRNA activity against miR-145-5p in PCa cells. This lncRNA is upregulated in PCa tissues and cell lines compared with normal prostate epithelial tissues and cells. By combining computational predictions with experimental validations, we demonstrated that lnc-ZNF30-3 contains five response elements that match canonical and non-canonical miR-145 seed sequences, and interacts with miR-145-5p in a seed sequence-dependent manner. Indeed, biotinylated miR-145-5p can pull-down lnc-ZNF30-3 and AGO2. This interaction may be abolished by two-point mutations in the miR-145-5p seed site, highlighting its specificity. SiRNA-mediated lnc-ZNF30-3 depletion of lnc-ZNF30-3 in PC3a cells results in reduced cell migration. Observed changes in migratory properties of cells are associated with a decrease in expression of mesenchymal transcription factors TWIST1 and ZEB1 at both the RNA and protein levels. These findings suggested that lnc-ZNF30-3 knockdown promotes a partial loss of mesenchymal traits to the benefit of a more pronounced epithelial phenotype.

In this context of low lnc-ZNF30-3 levels, further inhibition of miR-145-5p allows a rescue of cell motility and upregulation of TWIST1 and ZEB1. This indicates that the effect of lnc-ZNF30-3 depletion can be related to a release of miR-145-5p and its interaction with various functional targets such as TWIST1. Computational predictions and literature survey identified REs for 35 out of 36 miRNAs experimentally proven to target and control TWIST1 expression in various cancers. Dysregulated expression of 11 of these miRNAs was found in PCa, 9 being downregulated. This observation suggested that lnc-ZNF30-3 potentially works as a sponge for those miRNAs. It is important to mention that upregulation of lnc-ZNF30-3 was comparable to upregulation of TWIST1 in seven PCa versus normal tissues. Moreover, TWIST1 upregulation in PCa tissues from the TCGA cohort corelates with down-regulation of miR-145 expression, particularly during late-stage PCa progression. Notably, expression changes in TWIST1 and miR-145 expression in PCa cannot be fully explained by accumulation of genetic abnormalities during PCa development, leaving room for the involvement of epigenetic regulatory mechanisms. We uncovered for the first time lnc-ZNF30-3 sponge function to be an important regulatory mechanism. Furthermore, lnc-ZNF30-3 can deplete most miRNAs that already been proven to target and control other EMT transcription factors, such as TWIST2, ZEB1, ZEB2, SNAIL1, and SNAIL2 in various cancers. Cancer cells are characterized by high plasticity when they undergo the EMT process. Changes in EMT genes expression can disbalance and sense epithelial cells to a partial transition through intermediate, hybrid states towards progressive acquisition of mesenchymal properties. Our results suggest that lnc-ZNF30-3, by counteracting the activity of miR-145-5p and probably other microRNAs, may contribute to the increased expression of EMT factors, thereby allowing cancer cells to progress and metastasize.

Lnc-ZNF30-3 expression in PC3a cells is relatively low. Nevertheless, further depletion of lncRNA still had an effect on cell properties. It could be that expression of this lncRNA is heterogeneous among the tumor cell population. Additionally, lnc-ZNF30-3 could be secreted along with miR-145-5p as a part of extracellular vesicles. It has been shown that the miR-145-5p to be actively transported outside the cells in colorectal [37], lung [38], and prostate [39] cancers by a mechanism that has not yet been characterized. As a matter of fact, we observed an accumulation of lnc-ZNF30-3 and miR-145-5p outside proliferating and, to a lesser extent, in senescent fibroblasts (unpublished). Thus, the role of lnc-ZNF30-3 and miR-145-5p in cell-to-cell communication warrants further investigation.

Moreover, bioinformatics analysis revealed that in addition to miR-145, lnc-ZNF30-3 also contains potential binding sites to other miRNAs experimentally proven to target TWIST1, but also binding sites to most miRNAs that target other mesenchymal transcription factors, such as TWIST2, SNAIL1/2, and ZEB1/2 in various cancer models.

## Conclusions

We identified lnc-ZNF30-3 as a novel endogenous competing lncRNA for miR-145-5p and other miRNAs that target TWIST1 in prostate and other cancers. Our findings support its role in EMT endowing cancer cells with mesenchymal properties contributing to tumor progression and metastasis, and promote lnc-ZNF30-3 as a promising biomarker candidate for risk stratification, but also as a target in development of alternative PCa therapies.

## Electronic supplementary material

Below is the link to the electronic supplementary material.

Supplementary Material 1

## Data Availability

The data generated or analyzed during this study are included in this manuscript and its supplementary information files.

## Abbreviations

ACT1: Actin
as-lncRNA: antisense lncRNA
ceRNA: competing endogenous lncRNA
CHD1: E-Cadherin
EMT: Epithelial-to-Mesenchymal Transition
FN1: Fibronectin
FFPE: Formalin-Fixed Paraffin-Embedded
H&E: Hematoxylin and eosin
HGPIN: high-grade prostatic intraepithelial neoplasia
lncRNA: long non-coding RNA
PCa: Prostate Cancer
PRAD: PRostate ADenocarcinoma
so-lncRNA: sense overlapping lncRNA
TCGA: The Cancer Genome Atlas
VIM: Vimentin
vs.: versus
