# Supplementary material for: Pro-tumorigenic role of lnc-ZNF30-3 as a sponge counteracting miR-145-5p in prostate cancer
Source: Biol Direct. 2023 Jul 11;18:38. doi: 10.1186/s13062-023-00393-7 (PMC10334624; doi:10.1186/s13062-023-00393-7)
Supplement: Supplementary file 1 — Supplementary Material 1 [file 13062_2023_393_MOESM1_ESM.docx]

# Supplementary Materials

**Supplementary Figures:**

**Supplementary Figure 1.** Micro dissected FFPE tissues at different stages of PCa progression used in this study for miR-145 quantification.

**A**. Representative images of immunohistochemical staining to assess the expression of E-cadherin (epithelial marker), Vimentin (mesenchymal marker) and Ki67 (proliferation marker) in normal prostates and in PCa tissues at different stages of progression comprising high-grade prostatic intraepithelial neoplasia (HGPIN), invasive prostate cancer, and lymph node metastases. Hematoxylin and eosin (H&E) staining was applied for microdissection of the epithelial compartment for miRNA expression analysis.

**B**. Percentage of copy number alterations affecting the miR-145-5p (left) and TWIST1 (right) locus calculated with the use of cBioPortal across 10 different PCa cohorts (1). Cohorts: 1 - Prostate Cancer (MSKCC, JCO Precis Oncol 2017); 2 - Prostate Adenocarcinoma (MSKCC, PNAS 2014); 3 - Metastatic Prostate Adenocarcinoma (SU2C/PCF Dream Team, PNAS 2019); 4 - Metastatic Prostate Adenocarcinoma (MCTP, Nature 2012); 5 - Prostate Adenocarcinoma (TCGA, PanCancer Atlas); 6 - Prostate Adenocarcinoma (MSKCC, Cancer Cell 2010); 7 – Prostate Adenocarcinoma (Fred Hutchinson CRC, Nat Med 2016); 8 - The Metastatic Prostate Cancer Project (Provisional, June 2021); 9 - Prostate Adenocarcinoma (MSKCC/DFCI, Nature Genetics 2018); 10 – Metastatic Prostate Adenocarcinoma (SU2C/PCF Dream Team, Cell 2015).

**Supplementary Figure 2**. **Identification of miRNA REs proved to target TWIST1 3’UTR in different cancers**.

TWIST1 3’UTR (926-1634 nucleotides, [GeneBank: NM_011658]) with RE sequences in blue and underlined with corresponded miRNAs names.

**Supplementary Figure 3. Identification of REs of TWIST1 targeting miRNA within lnc-ZNF30-3.**

Lnc-ZNF30-3 sequence (5394 nucleotides, ENST00000601776.2) with RE sequences in blue and underlined with corresponded miRNA names.

**Supplementary Tables:**

**Supplementary Table 1.** Clinicopathological characteristics of the FFPE cohort of PCa tissues.

**Supplementary Table 2.** Primers used in this study for qPCR.

**Supplementary Table 3.** Number of putative REs for miR-145-5p within lnc-ZNF30-3 identified by seed match analysis.

**Supplementary Table 4.** MiRNAs experimentally proved to bind and regulate expression of TWIST1, and corresponding REs identified within the lnc-ZNF30-3 transcript.

**Supplementary Table 5.** Number of REs for miRNAs experimentally proved to bind and regulate expression of EMT factors: TWIST1/2, ZEB1/2, SNAIL1/2, and putative REs within lnc-ZNF30-3

**Supplementary Figure 1**


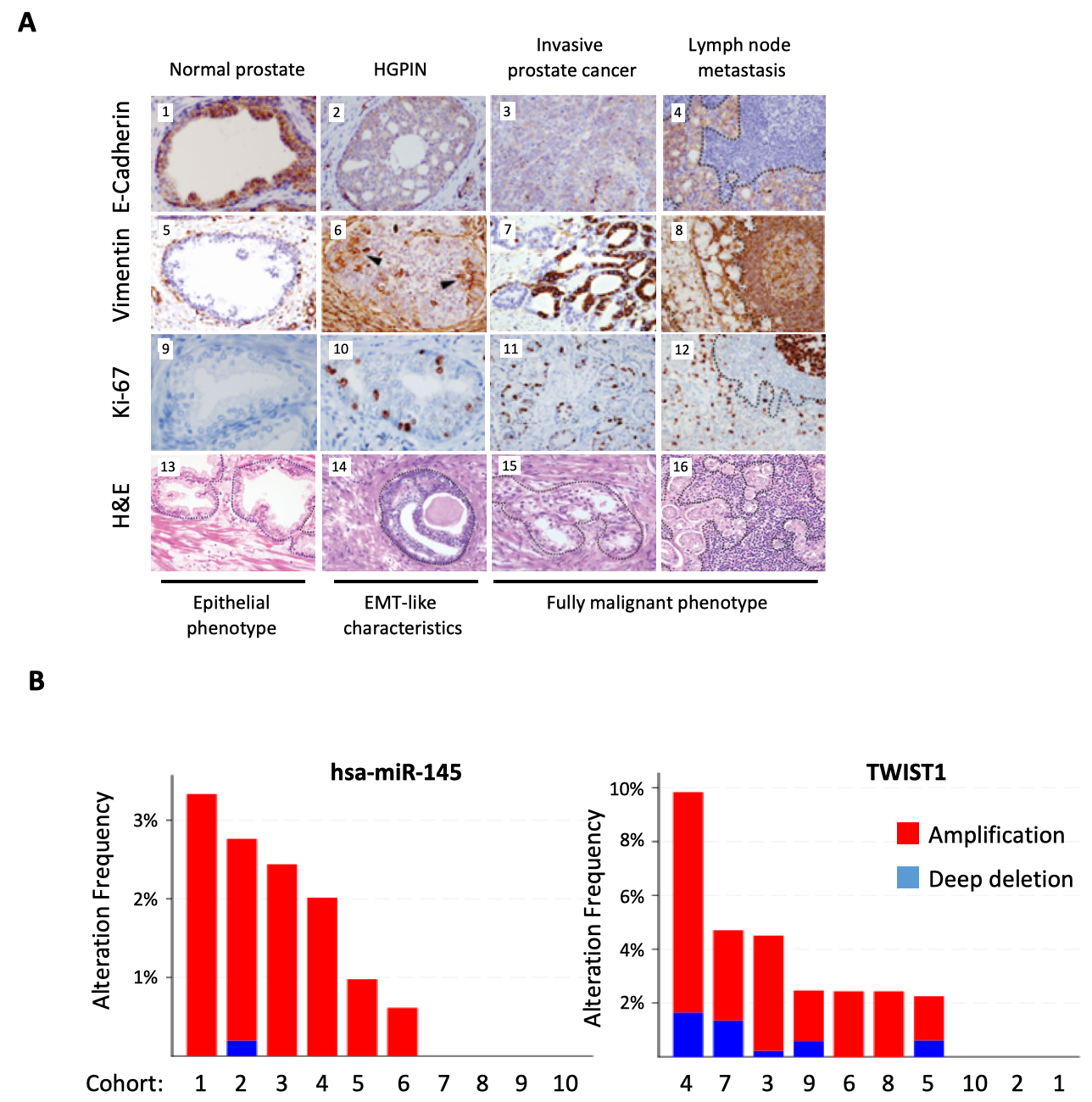


**Supplementary Figure 2**


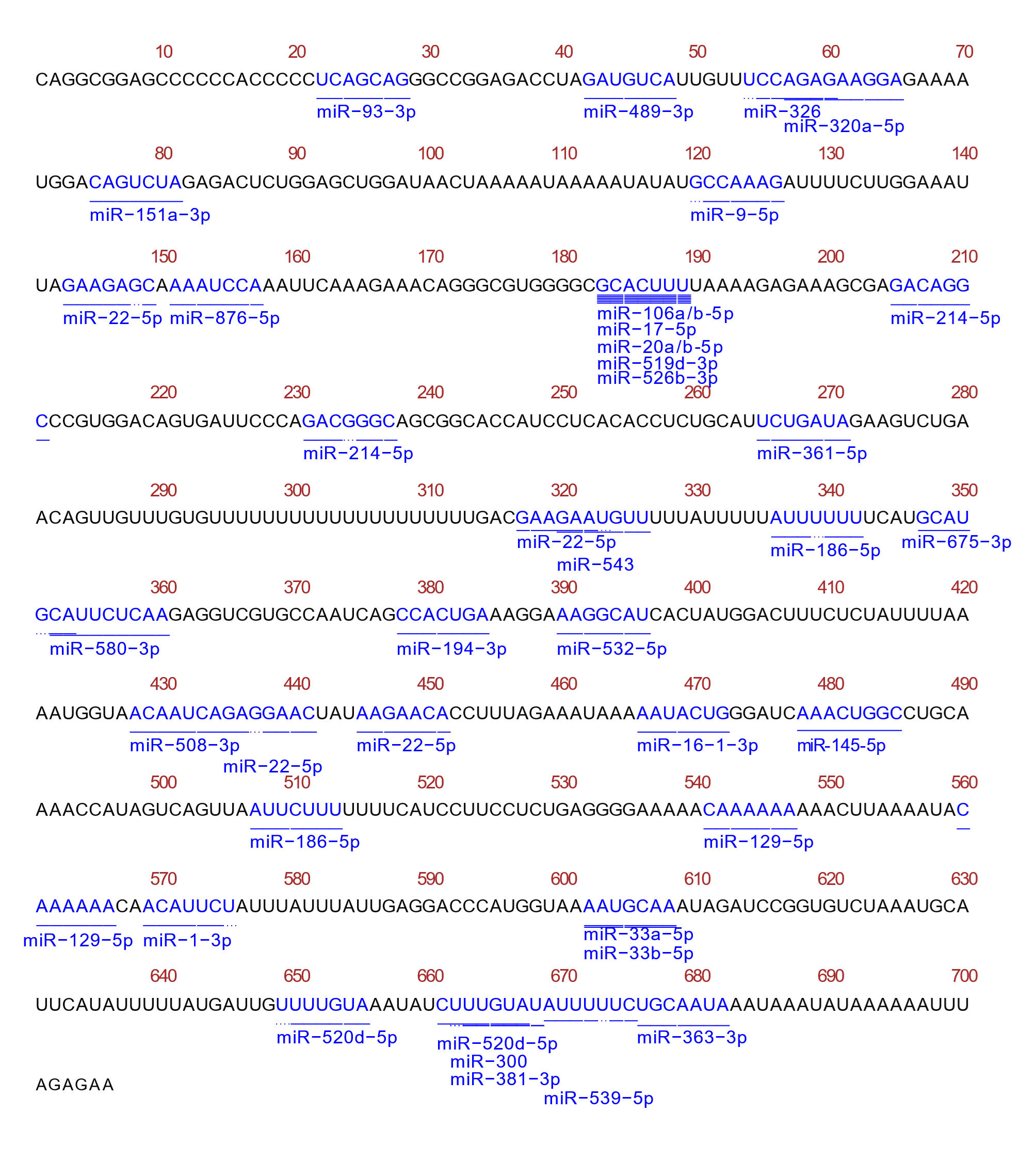


**Supplementary Figure 3**


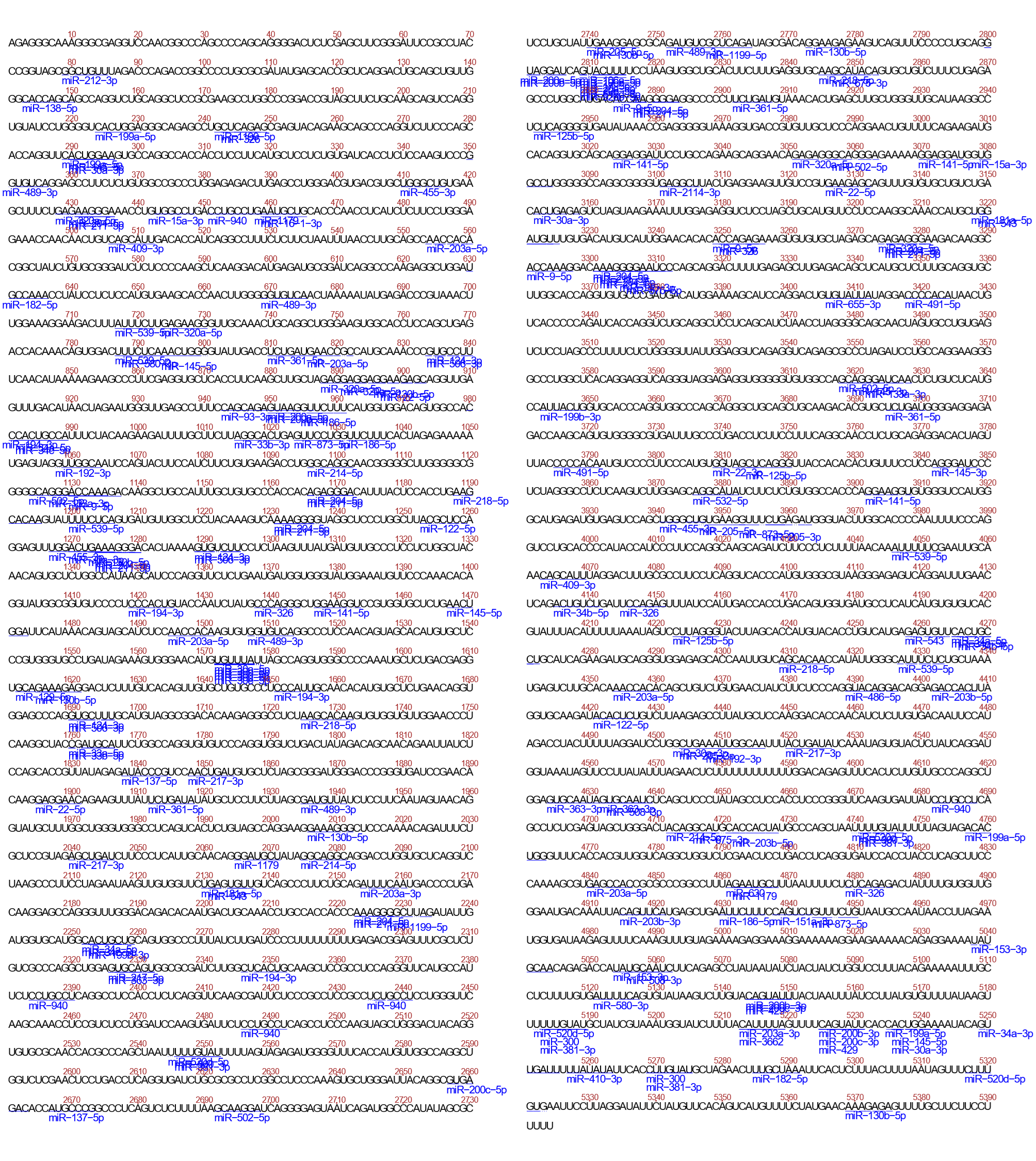


**Supplementary Table 1. Clinicopathological characteristics of the FFPE cohort of PCa tissues**

| **Patient** | **Age at diagnosis (yr)** | **Gleason score** | **PSA at diagnosis**  **(ng/ml)** | **D’Amico Classification** | **Surgical margin** | **TNM** | **Treatment** | **PSA afer RP** |
| --- | --- | --- | --- | --- | --- | --- | --- | --- |
| **1** | 43 | 3+3=6 | 2.6 | Low | Positive | pT2cPNXM0 | RP | 0.07 |
| **2** | 42 | 3+3=6 | 10.8 | Low | Positive | pT2cpN0M0 | RP + LY. RA | 0.05 |
| **3** | 56 | 3+3=6 | 4.8 | Low | Negative | pT2cPNXM0 | RP + RA | Undetectable |
| **4** | 46 | 3+3=6 | 6.5 | Low | Negative | pT2cPNXM0 | RP | 0.03 |
| **5** | 64 | 3+4=7 | 6.99 | Intermediate | Negative | pT2cpN0M0 | RP + LY | 0.002 |
| **6** | 64 | 3+4=7 | 4.8 | Intermediate | Positive | pT2cpN0M0 | RP + LY. RA | 0.03 |
| **7** | 67 | 3+4=7 | 8 | Intermediate | Positive | pT2cPNXM0 | RP | 0.07 |
| **8** | 59 | 3+4=7 | 1.9 | Intermediate | Negative | pT2cPNXM0 | RP | 0.04 |
| **9** | 50 | 3+4=7 | 31 | High | Negative | pT3N0M0 | RP + LY. Androgen deprivation therapy | 1.52 |
| **10** | 61 | 4+3=7 | 12.5 | High | Negative | pT3bpNOM0 | RP + LY Dietibestrol RA  Bicalutamide, Docetaxel  Abiraterone | 2.6 |
| **11** | 67 | 3+4=7 | 4.6 | High | Positive | pT2cpN0M0 | RP + LY Dietibestrol | 0.07 |
| **12** | 55 | 4+3=7 | 3.8 | High | Positive | T2bN0M | RP + LY. RA | 0.03 |
| **13** | 53 | 4+3=7+5 | 15,1 | High | Positive | pT2cN1M0. | RP + LY | N.A. |
| **14** | 57 | 4+3=7 | 13 | High | Positive | pT2cpN0M0 | RP + LY hormotherapy  (Goserelin) | 15.9 |
| **15** | 66 | 4+3=7 | 10.2 | High | Positive | T2bN0M | RP + LY  Hormotherapy  (Leuprolide) | N.A. |
| **16** | 70 | 4+5=9 | 5.09 | High | Negative | pT2cN1M0. | RP + LY | N.A. |

PSA, prostate specific antigen; TNM, Tumor Node Metastasis; RP, Radical prostatectomy; LY, pelvic lymph node dissection; RA: radiotherapy; N.A. not available

**Supplementary Table 2. QPCR-primers**

| VIM (Vimentin) | Forward: CGAGGACGAGGAGAGCAGGATTTCTC Reverse: GGTATCAACCAGAGGGAGTGA |
| --- | --- |
| CHD1 (E-cadherin) | Forward: TGGACAGGGAGGATTTTGAG Reverse: ACCCACCTCTAAGGCCATCT |
| SNAI1 | Forward: ACCACTATGCCGCGCTCTT Reverse: GGTCGTAGGGCTGCTGGAA |
| SNAI2(Slug) | Forward: TGTTGCAGTGAGGGCAAGAA Reverse: GACCCTGGTTGCTTCAAGGA |
| TWIST1 | Forward: GCCAGGTACATCGACTTCCTCT Reverse: TCCATCCTCCAGACCGAGAAGG |
| ZEB1 | Forward: GGCATACACCTACTCAACTACGG Reverse: TGGGCGGTGTAGAATCAGAGTC |
| FN1 | Forward: GGCATACACCTACTCAACTACGG Reverse: TGGGCGGTGTAGAATCAGAGTC |
| MMP3 | Forward: CACTCACAGACCTGACTCGGTT Reverse: AAGCAGGATCACAGTTGGCTGG |
| Lnc-ZNF30-3 | Forward: ATGTAGGCGGACACAAGAGG Reverse: ATCCCGCTAGAGCACATCAG |
| PRNCR1 | Forward: CCAGATTCCAAGGGCTGATA Reverse: GATGTTTGGAGGCATCTGGT |
| DLX6-AS1 | Forward: AGACAAAGGAAAACCCACTCC Reverse: TGATGATGGTGTCCAGGAGC |
| AC004947.2 | Forward: GGCAACACAGTTTATATCATCTG Reverse: CATCAACTGGTCATTCGACATG |
| Lnc -NF30-3.2 | Forward: CCAACCTCATCTCTTCCTGG Reverse: ACAGATAGCCGTGTGGTTGG |
| ACTB/ACTG1 | Forward: CACCATTGGCAATGAGCGGTTC Reverse: AGGTCTTTGCGGATGTCCACGT |
| GAPDH | Forward: TGGGCTACACTGAGCACCAG Reverse: CAGCGTCAAAGGTGGAGGAG |

**Supplementary Table 3 PCa upregulated LncRNAs** ( LncRNAs associated with PCa in previous studies are labeled in red)

| id | Name | Type | FC | rawp | Number of miR-145-5p REs |
| --- | --- | --- | --- | --- | --- |
| ENSG00000282961.1_2 | PRNCR1 | lincRNA | 4.5154 | 0.000 | 6 |
| ENSG00000231764.8_2 | DLX6-AS1 | antisense | 3.4889 | 0.002 | 6 |
| ENSG00000281778.1_2 | LINC00550 | lincRNA | 46.4947 | 0.000 | 5 |
| ENSG00000233878.1_2 | AC073133.1 | lincRNA | 5.2754 | 0.000 | 4 |
| ENSG00000261146.1_2 | RP11-2A4.4 | lincRNA | 6.0418 | 0.000 | 4 |
| ENSG00000269086.2_2 | lnc-ZNF30-3 | lincRNA | 8.0158 | 0.000 | 4 |
| ENSG00000233221.6_2 | AC133785.1 | antisense | 4.6291 | 0.001 | 4 |
| ENSG00000235296.1_2 | AC137723.5 | antisense | 3.5636 | 0.003 | 4 |
| ENSG00000261324.2_2 | RP11-174G6.5 | sense_overlapping | 2.4938 | 0.003 | 4 |
| ENSG00000122548.5_2 | KIAA0087 | lincRNA | 20.5893 | 0.000 | 3 |
| ENSG00000182366.9_2 | FAM87A | lincRNA | 9.5041 | 0.000 | 3 |
| ENSG00000237352.3_2 | LINC01358 | lincRNA | 3.6083 | 0.000 | 3 |
| ENSG00000260896.5_2 | LINC02170 | lincRNA | 8.4081 | 0.000 | 3 |
| ENSG00000280719.1_2 | PCAT5 | lincRNA | 19.9396 | 0.000 | 3 |
| ENSG00000282998.1_2 | RP11-152D8.1 | lincRNA | 2.9841 | 0.001 | 3 |
| ENSG00000236445.4_2 | LINC00608 | antisense | 5.8091 | 0.000 | 3 |
| ENSG00000273523.1_2 | RP11-248G5.9 | antisense | 7.3988 | 0.000 | 3 |
| ENSG00000215158.9_3 | RP11-1023L17.1 | transcribed_unprocessed_pseudogene | 3.7599 | 0.000 | 3 |
| ENSG00000172250.14_2 | SERHL | processed_transcript | 2.9389 | 0.001 | 3 |
| ENSG00000217576.7_3 | RP11-248G5.8 | processed_transcript | 5.9378 | 0.000 | 3 |
| ENSG00000181908.5_2 | AP003774.4 | lincRNA | 4.8479 | 0.001 | 2 |
| ENSG00000213468.4_3 | FIRRE | lincRNA | 4.8677 | 0.000 | 2 |
| ENSG00000223838.1_2 | AC007091.1 | lincRNA | 37.0947 | 0.000 | 2 |
| ENSG00000224899.1_2 | RP11-3B12.5 | lincRNA | 4.7101 | 0.002 | 2 |
| ENSG00000225206.8_3 | MIR137HG | lincRNA | 45.2732 | 0.000 | 2 |
| ENSG00000232806.1_2 | AP001610.9 | lincRNA | 24.0914 | 0.000 | 2 |
| ENSG00000233760.1_2 | AC004947.2 | lincRNA | 16.2060 | 0.000 | 2 |
| ENSG00000236081.1_2 | ELFN1-AS1 | lincRNA | 6.0194 | 0.000 | 2 |
| ENSG00000246898.1_2 | LINC00920 | lincRNA | 4.2656 | 0.000 | 2 |
| ENSG00000248515.1_3 | RP11-608O21.1 | lincRNA | 10.9146 | 0.000 | 2 |
| ENSG00000249267.6_2 | LINC00939 | lincRNA | 5.1356 | 0.000 | 2 |
| ENSG00000260951.1_2 | RP5-978I12.1 | lincRNA | 3.7774 | 0.000 | 2 |
| ENSG00000272732.1_2 | RP5-1159O4.2 | lincRNA | 5.4340 | 0.000 | 2 |
| ENSG00000231150.5_2 | RP1-207H1.3 | antisense | 3.1049 | 0.000 | 2 |
| ENSG00000246228.6_2 | CASC8 | antisense | 5.0726 | 0.000 | 2 |
| ENSG00000262768.2_2 | RP11-353N14.1 | antisense | 3.7523 | 0.001 | 2 |
| ENSG00000266441.1_2 | RP11-91I8.3 | antisense | 6.9132 | 0.000 | 2 |
| ENSG00000251643.1_2 | RP11-91J3.1 | processed_pseudogene | 3.9219 | 0.002 | 2 |
| ENSG00000243316.7_2 | GUCY2GP | transcribed_unitary_pseudogene | 6.6041 | 0.000 | 2 |
| ENSG00000283380.1_2 | RP11-274G22.1 | processed_transcript | 7.7938 | 0.000 | 2 |
| ENSG00000203635.2_2 | AC144450.2 | lincRNA | 18.4481 | 0.000 | 1 |
| ENSG00000223715.1_2 | LINC01208 | lincRNA | 5.1932 | 0.001 | 1 |
| ENSG00000224506.2_2 | RP1-293L8.2 | lincRNA | 6.3849 | 0.000 | 1 |
| ENSG00000229403.1_2 | RP4-718N17.2 | lincRNA | 8.0056 | 0.000 | 1 |
| ENSG00000231010.1_2 | RP6-109B7.2 | lincRNA | 7.0525 | 0.000 | 1 |
| ENSG00000232353.5_2 | RP11-655G22.1 | lincRNA | 4.9349 | 0.002 | 1 |
| ENSG00000232677.6_2 | LINC00665 | lincRNA | 2.6522 | 0.002 | 1 |
| ENSG00000233056.2_2 | ERVH48-1 | lincRNA | 6.1975 | 0.000 | 1 |
| ENSG00000233080.2_3 | LINC01399 | lincRNA | 4.3217 | 0.000 | 1 |
| ENSG00000233215.5_2 | LINC01687 | lincRNA | 6.8896 | 0.000 | 1 |
| ENSG00000233515.1_2 | LINC01518 | lincRNA | 21.3654 | 0.000 | 1 |
| ENSG00000235091.1_2 | WI2-85898F10.1 | lincRNA | 6.7698 | 0.000 | 1 |
| ENSG00000235140.1_2 | RP11-135D11.2 | lincRNA | 12.3572 | 0.000 | 1 |
| ENSG00000236030.1_2 | LINC01036 | lincRNA | 2.8339 | 0.001 | 1 |
| ENSG00000237612.1_2 | AP002856.7 | lincRNA | 5.1442 | 0.002 | 1 |
| ENSG00000237838.1_2 | AC133680.1 | lincRNA | 2.2959 | 0.001 | 1 |
| ENSG00000241098.1_3 | LINC01994 | lincRNA | 8.1727 | 0.000 | 1 |
| ENSG00000243694.2_2 | LINC02027 | lincRNA | 5.8316 | 0.001 | 1 |
| ENSG00000248131.5_3 | LINC01194 | lincRNA | 9.1777 | 0.000 | 1 |
| ENSG00000248874.5_3 | C5orf17 | lincRNA | 12.7021 | 0.000 | 1 |
| ENSG00000249859.9_3 | PVT1 | lincRNA | 2.2379 | 0.001 | 1 |
| ENSG00000253264.1_2 | PCAT2 | lincRNA | 4.7513 | 0.002 | 1 |
| ENSG00000253438.2_2 | PCAT1 | lincRNA | 3.2443 | 0.000 | 1 |
| ENSG00000254349.5_3 | MIR2052HG | lincRNA | 2.1227 | 0.001 | 1 |
| ENSG00000254573.1_2 | AP004550.1 | lincRNA | 4.7475 | 0.001 | 1 |
| ENSG00000256276.1_3 | RP5-916L7.2 | lincRNA | 4.1310 | 0.002 | 1 |
| ENSG00000258479.5_2 | LINC00640 | lincRNA | 6.6946 | 0.000 | 1 |
| ENSG00000258798.1_3 | RP11-895M11.3 | lincRNA | 5.1408 | 0.000 | 1 |
| ENSG00000259641.5_2 | PCAT29 | lincRNA | 3.4961 | 0.000 | 1 |
| ENSG00000261211.1_2 | RP1-80N2.3 | lincRNA | 5.8078 | 0.000 | 1 |
| ENSG00000264448.5_3 | RP3-388N13.5 | lincRNA | 4.4192 | 0.001 | 1 |
| ENSG00000265933.5_2 | LINC00668 | lincRNA | 10.3745 | 0.000 | 1 |
| ENSG00000266290.1_2 | RP11-159D12.10 | lincRNA | 4.6232 | 0.000 | 1 |
| ENSG00000271850.1_2 | RP11-16D22.2 | lincRNA | 3.6747 | 0.000 | 1 |
| ENSG00000272894.5_2 | RP5-1159O4.1 | lincRNA | 4.9847 | 0.000 | 1 |
| ENSG00000279712.1_2 | CTA-280A3.2 | lincRNA | 16.6198 | 0.000 | 1 |
| ENSG00000284418.1_1 | RP11-370B11.4 | lincRNA | 3.4763 | 0.000 | 1 |
| ENSG00000083622.8_2 | AC000111.6 | antisense | 8.3657 | 0.000 | 1 |
| ENSG00000204832.9_2 | ST8SIA6-AS1 | antisense | 8.6912 | 0.000 | 1 |
| ENSG00000225792.1_2 | AC004540.4 | antisense | 11.1591 | 0.000 | 1 |
| ENSG00000234715.1_2 | CTB-107G13.1 | antisense | 7.0114 | 0.000 | 1 |
| ENSG00000235749.2_3 | RP11-634B7.4 | antisense | 3.2579 | 0.000 | 1 |
| ENSG00000236283.4_3 | AC013463.2 | antisense | 4.1183 | 0.000 | 1 |
| ENSG00000248429.5_3 | RP11-597D13.9 | antisense | 4.2290 | 0.000 | 1 |
| ENSG00000249494.5_2 | CTB-161M19.4 | antisense | 4.6167 | 0.000 | 1 |
| ENSG00000250910.7_3 | AC097467.2 | antisense | 14.6948 | 0.000 | 1 |
| ENSG00000254680.1_2 | RP11-265D17.2 | antisense | 5.5271 | 0.000 | 1 |
| ENSG00000254988.1_2 | CTD-2547H18.1 | antisense | 6.7185 | 0.000 | 1 |
| ENSG00000255240.5_3 | RP11-142C4.6 | antisense | 4.6794 | 0.000 | 1 |
| ENSG00000261373.1_2 | VPS9D1-AS1 | antisense | 4.4138 | 0.001 | 1 |
| ENSG00000266711.1_2 | RP11-398J5.1 | antisense | 5.0207 | 0.000 | 1 |
| ENSG00000268686.1_2 | AC010524.2 | antisense | 4.7514 | 0.001 | 1 |
| ENSG00000273179.1_2 | RP11-20I20.4 | antisense | 4.7445 | 0.001 | 1 |
| ENSG00000278907.1_2 | AC005609.19 | antisense | 5.2344 | 0.000 | 1 |
| ENSG00000280927.1_2 | CTBP1-AS | antisense | 12.0460 | 0.000 | 1 |
| ENSG00000219736.1_2 | RP11-560O20.1 | processed_pseudogene | 5.9118 | 0.000 | 1 |
| ENSG00000225557.1_3 | MTCO3P20 | processed_pseudogene | 7.3742 | 0.000 | 1 |
| ENSG00000229088.1_2 | MTND1P10 | processed_pseudogene | 7.1145 | 0.000 | 1 |
| ENSG00000229332.2_2 | PGBD4P8 | processed_pseudogene | 21.4974 | 0.000 | 1 |
| ENSG00000230335.1_2 | DNAJB5P1 | processed_pseudogene | 3.3669 | 0.001 | 1 |
| ENSG00000230376.1_2 | MEMO1P4 | processed_pseudogene | 3.3347 | 0.000 | 1 |
| ENSG00000233764.1_2 | MTCO1P20 | processed_pseudogene | 7.6943 | 0.000 | 1 |
| ENSG00000237129.1_2 | MTCYBP34 | processed_pseudogene | 11.2648 | 0.000 | 1 |
| ENSG00000248822.1_2 | APOBEC3AP1 | processed_pseudogene | 8.3114 | 0.000 | 1 |
| ENSG00000271013.1_2 | LRRC37A9P | processed_pseudogene | 3.5667 | 0.002 | 1 |
| ENSG00000234381.1 | MED15P7 | pseudogene | 7.4372 | 0.000 | 1 |
| ENSG00000230246.7_2 | SPATA31C1 | transcribed_unprocessed_pseudogene | 5.1790 | 0.001 | 1 |
| ENSG00000234493.3_2 | RHOXF1P1 | transcribed_unprocessed_pseudogene | 6.7439 | 0.000 | 1 |
| ENSG00000237438.7_3 | CECR7 | transcribed_unprocessed_pseudogene | 2.8361 | 0.002 | 1 |
| ENSG00000241549.8_2 | GUSBP2 | transcribed_unprocessed_pseudogene | 2.4285 | 0.001 | 1 |
| ENSG00000241388.4_2 | HNF1A-AS1 | processed_transcript | 5.5417 | 0.000 | 1 |
| ENSG00000223523.1_3 | AC079613.1 | sense_overlapping | 17.3843 | 0.000 | 1 |
| ENSG00000284500.1_1 | RP11-69H14.6 | sense_overlapping | 7.7138 | 0.000 | 1 |
| ENSG00000132204.13_3 | LINC00470 | lincRNA | 4.4467 | 0.000 | 0 |
| ENSG00000182648.11_2 | LINC01006 | lincRNA | 4.2028 | 0.000 | 0 |
| ENSG00000185044.10 | RP11-435B5.4 | lincRNA | 2.8901 | 0.000 | 0 |
| ENSG00000205628.2_2 | LINC01446 | lincRNA | 4.9951 | 0.000 | 0 |
| ENSG00000214691.7_2 | LINC01913 | lincRNA | 10.1513 | 0.000 | 0 |
| ENSG00000214870.8_2 | AC004540.5 | lincRNA | 4.1859 | 0.000 | 0 |
| ENSG00000223400.1_2 | AP006748.1 | lincRNA | 27.8104 | 0.000 | 0 |
| ENSG00000223477.3 | LINC00842 | lincRNA | 2.5555 | 0.001 | 0 |
| ENSG00000223930.5_2 | RP11-33A14.1 | lincRNA | 3.8993 | 0.000 | 0 |
| ENSG00000224141.5_2 | MIR548XHG | lincRNA | 6.1031 | 0.001 | 0 |
| ENSG00000224271.5_2 | RP11-191L9.4 | lincRNA | 20.9897 | 0.000 | 0 |
| ENSG00000224322.1_3 | AC004009.3 | lincRNA | 5.1512 | 0.000 | 0 |
| ENSG00000224330.1_2 | AC005019.3 | lincRNA | 6.4427 | 0.001 | 0 |
| ENSG00000224477.5_2 | RP1-81D8.3 | lincRNA | 4.2687 | 0.000 | 0 |
| ENSG00000224932.1_2 | AC107399.2 | lincRNA | 5.6711 | 0.001 | 0 |
| ENSG00000225258.1_3 | AC009478.1 | lincRNA | 2.9312 | 0.000 | 0 |
| ENSG00000225680.1_2 | AL163953.2 | lincRNA | 5.1481 | 0.001 | 0 |
| ENSG00000225882.1_2 | LINC01456 | lincRNA | 45.8509 | 0.000 | 0 |
| ENSG00000226383.6_3 | LINC01876 | lincRNA | 2.4097 | 0.000 | 0 |
| ENSG00000226825.1_2 | LINC01509 | lincRNA | 7.3178 | 0.000 | 0 |
| ENSG00000226906.1_2 | TTTY4 | lincRNA | 4.0642 | 0.001 | 0 |
| ENSG00000227342.1_2 | LINC00307 | lincRNA | 5.0609 | 0.000 | 0 |
| ENSG00000227681.5_3 | RP11-307P5.1 | lincRNA | 4.3457 | 0.000 | 0 |
| ENSG00000228538.5_2 | AC009411.1 | lincRNA | 4.7313 | 0.000 | 0 |
| ENSG00000228559.1_2 | RP3-340B19.3 | lincRNA | 9.0522 | 0.000 | 0 |
| ENSG00000228566.1_2 | RP11-170M17.1 | lincRNA | 5.3592 | 0.000 | 0 |
| ENSG00000228569.1_2 | AC073133.2 | lincRNA | 4.4016 | 0.001 | 0 |
| ENSG00000228933.7_3 | RP11-268G12.1 | lincRNA | 14.5757 | 0.000 | 0 |
| ENSG00000230205.1_2 | RP11-631F7.2 | lincRNA | 3.7101 | 0.002 | 0 |
| ENSG00000230345.1_2 | RP13-455A7.1 | lincRNA | 10.1916 | 0.000 | 0 |
| ENSG00000230880.2 | RP11-417J8.3 | lincRNA | 4.5307 | 0.000 | 0 |
| ENSG00000231648.1_2 | LINC01698 | lincRNA | 5.0596 | 0.001 | 0 |
| ENSG00000231808.2_2 | LINC01388 | lincRNA | 9.1874 | 0.000 | 0 |
| ENSG00000232590.1_2 | RP11-128I7.1 | lincRNA | 4.3733 | 0.000 | 0 |
| ENSG00000233577.6_2 | RP3-462D8.2 | lincRNA | 3.6495 | 0.002 | 0 |
| ENSG00000234182.1_2 | RP11-118K6.2 | lincRNA | 5.5641 | 0.001 | 0 |
| ENSG00000234692.1_2 | RP11-445L6.3 | lincRNA | 8.4436 | 0.000 | 0 |
| ENSG00000234948.1_3 | LINC01524 | lincRNA | 3.8875 | 0.002 | 0 |
| ENSG00000235056.1_2 | AC010983.1 | lincRNA | 3.4328 | 0.000 | 0 |
| ENSG00000235159.1_2 | RP6-109B7.4 | lincRNA | 4.1924 | 0.000 | 0 |
| ENSG00000235533.1_2 | RP11-284P20.3 | lincRNA | 5.3333 | 0.000 | 0 |
| ENSG00000236651.1_2 | DLX2-AS1 | lincRNA | 8.1108 | 0.000 | 0 |
| ENSG00000236924.1_3 | RP11-390F4.6 | lincRNA | 4.8919 | 0.000 | 0 |
| ENSG00000237643.1_2 | RP11-462G2.1 | lincRNA | 12.8152 | 0.000 | 0 |
| ENSG00000237670.1_2 | LINC01866 | lincRNA | 15.6731 | 0.000 | 0 |
| ENSG00000237713.1_2 | AC006000.5 | lincRNA | 17.3549 | 0.000 | 0 |
| ENSG00000238261.5 | RP11-435B5.5 | lincRNA | 4.4172 | 0.002 | 0 |
| ENSG00000239513.5_2 | LINC01210 | lincRNA | 5.3646 | 0.000 | 0 |
| ENSG00000242012.1_2 | RP11-338L18.1 | lincRNA | 7.2264 | 0.000 | 0 |
| ENSG00000242021.2_3 | RP11-268G12.3 | lincRNA | 6.0747 | 0.000 | 0 |
| ENSG00000242781.1_2 | LINC02050 | lincRNA | 37.9079 | 0.000 | 0 |
| ENSG00000242828.1_2 | RP11-47P18.1 | lincRNA | 19.0921 | 0.000 | 0 |
| ENSG00000243479.3_2 | MNX1-AS1 | lincRNA | 15.2463 | 0.000 | 0 |
| ENSG00000245532.7_3 | NEAT1 | lincRNA | 2.1712 | 0.001 | 0 |
| ENSG00000245750.7_2 | DRAIC | lincRNA | 4.6328 | 0.000 | 0 |
| ENSG00000247011.2_2 | RP11-700H6.1 | lincRNA | 4.4656 | 0.000 | 0 |
| ENSG00000248143.1_2 | RP11-324J13.2 | lincRNA | 11.0896 | 0.000 | 0 |
| ENSG00000248279.5_2 | LINC02120 | lincRNA | 6.8296 | 0.000 | 0 |
| ENSG00000248663.6_2 | LINC00992 | lincRNA | 5.3458 | 0.000 | 0 |
| ENSG00000248927.1_2 | CTD-2334D19.1 | lincRNA | 6.4328 | 0.000 | 0 |
| ENSG00000249413.2_3 | RP11-25H12.1 | lincRNA | 12.0884 | 0.000 | 0 |
| ENSG00000249877.1_2 | RP11-706F1.2 | lincRNA | 4.5659 | 0.002 | 0 |
| ENSG00000249951.1_2 | RP11-554D13.1 | lincRNA | 6.4160 | 0.000 | 0 |
| ENSG00000250387.2_3 | LINC02197 | lincRNA | 10.7382 | 0.000 | 0 |
| ENSG00000250590.5_2 | RP11-565A3.2 | lincRNA | 6.1243 | 0.000 | 0 |
| ENSG00000251026.1_2 | LINC02163 | lincRNA | 6.3507 | 0.001 | 0 |
| ENSG00000251574.6_3 | RP11-6N13.1 | lincRNA | 4.7352 | 0.001 | 0 |
| ENSG00000253400.1_2 | RP11-337A23.6 | lincRNA | 21.5714 | 0.000 | 0 |
| ENSG00000253471.1_2 | RP11-175E9.1 | lincRNA | 3.8296 | 0.000 | 0 |
| ENSG00000253929.1_2 | CASC21 | lincRNA | 3.6665 | 0.000 | 0 |
| ENSG00000255693.1_2 | RP11-766N7.3 | lincRNA | 3.3860 | 0.001 | 0 |
| ENSG00000256513.1_2 | RP11-977P2.1 | lincRNA | 5.3507 | 0.002 | 0 |
| ENSG00000257268.1_2 | RP1-74B13.2 | lincRNA | 7.2547 | 0.000 | 0 |
| ENSG00000257582.5_2 | LINC01475 | lincRNA | 13.5167 | 0.000 | 0 |
| ENSG00000257955.1_2 | RP1-228P16.5 | lincRNA | 3.8045 | 0.002 | 0 |
| ENSG00000257989.1_2 | RP1-288H2.2 | lincRNA | 2.2171 | 0.001 | 0 |
| ENSG00000258710.7_3 | LINC01193 | lincRNA | 10.0643 | 0.000 | 0 |
| ENSG00000259087.5_2 | RP11-356O9.2 | lincRNA | 2.9626 | 0.002 | 0 |
| ENSG00000259457.1_2 | RP11-279F6.2 | lincRNA | 4.2836 | 0.000 | 0 |
| ENSG00000259471.1_2 | LINC01169 | lincRNA | 6.2951 | 0.000 | 0 |
| ENSG00000259946.1_2 | RP11-490G2.2 | lincRNA | 4.5552 | 0.001 | 0 |
| ENSG00000260209.1_2 | RP11-680F20.10 | lincRNA | 6.4047 | 0.000 | 0 |
| ENSG00000262585.1_2 | LINC01979 | lincRNA | 4.6773 | 0.002 | 0 |
| ENSG00000265369.3_2 | PCAT18 | lincRNA | 10.0547 | 0.000 | 0 |
| ENSG00000265717.1_2 | RP11-94B19.7 | lincRNA | 6.0977 | 0.001 | 0 |
| ENSG00000266554.1_2 | LINC01443 | lincRNA | 21.7624 | 0.000 | 0 |
| ENSG00000267501.1_2 | RP11-108P20.2 | lincRNA | 7.7090 | 0.000 | 0 |
| ENSG00000267705.1_3 | RP11-108P20.3 | lincRNA | 7.5940 | 0.000 | 0 |
| ENSG00000270141.3_2 | TERC | lincRNA | 3.3151 | 0.000 | 0 |
| ENSG00000270372.1_2 | RP11-109M17.2 | lincRNA | 12.1056 | 0.000 | 0 |
| ENSG00000271893.1_2 | RP11-762E8.1 | lincRNA | 6.8702 | 0.000 | 0 |
| ENSG00000272108.1_2 | AC005754.8 | lincRNA | 4.7877 | 0.001 | 0 |
| ENSG00000272243.5_3 | RP11-554D15.3 | lincRNA | 7.2840 | 0.000 | 0 |
| ENSG00000272662.1_2 | RP11-190C22.8 | lincRNA | 3.6073 | 0.002 | 0 |
| ENSG00000274685.1_2 | RP11-122K13.15 | lincRNA | 5.7628 | 0.001 | 0 |
| ENSG00000274979.1_2 | RP11-1143G9.5 | lincRNA | 10.3871 | 0.000 | 0 |
| ENSG00000278041.1_2 | RP5-984P4.6 | lincRNA | 14.6867 | 0.000 | 0 |
| ENSG00000280366.1_2 | RP11-327L9.1 | lincRNA | 4.0598 | 0.002 | 0 |
| ENSG00000280623.1_2 | PCAT14 | lincRNA | 6.2941 | 0.000 | 0 |
| ENSG00000280724.1_2 | RP11-11H9.2 | lincRNA | 6.7885 | 0.000 | 0 |
| ENSG00000281131.1_2 | SCHLAP1 | lincRNA | 8.3504 | 0.000 | 0 |
| ENSG00000281566.2_2 | RP11-485F13.1 | lincRNA | 6.4132 | 0.001 | 0 |
| ENSG00000176349.11_2 | AC110781.3 | antisense | 4.4256 | 0.001 | 0 |
| ENSG00000223392.1_2 | CLDN10-AS1 | antisense | 35.7698 | 0.000 | 0 |
| ENSG00000224063.5_3 | AC007319.1 | antisense | 2.4136 | 0.001 | 0 |
| ENSG00000224269.1_2 | AP000697.6 | antisense | 6.5167 | 0.000 | 0 |
| ENSG00000225652.1_2 | KCNMA1-AS3 | antisense | 4.3417 | 0.002 | 0 |
| ENSG00000225937.1_2 | PCA3 | antisense | 46.3688 | 0.000 | 0 |
| ENSG00000226622.5_2 | AC092155.4 | antisense | 6.0296 | 0.000 | 0 |
| ENSG00000226779.1_2 | NAALADL2-AS2 | antisense | 173.6563 | 0.000 | 0 |
| ENSG00000227695.5_2 | DNMBP-AS1 | antisense | 3.6439 | 0.001 | 0 |
| ENSG00000228113.7_3 | AC003991.3 | antisense | 3.4193 | 0.002 | 0 |
| ENSG00000228613.1_3 | AC144450.1 | antisense | 12.5588 | 0.000 | 0 |
| ENSG00000229751.1_2 | RP11-142M10.2 | antisense | 6.8967 | 0.000 | 0 |
| ENSG00000230061.2_2 | TRPM2-AS | antisense | 8.8582 | 0.000 | 0 |
| ENSG00000230798.5_2 | FOXD3-AS1 | antisense | 4.7934 | 0.001 | 0 |
| ENSG00000231061.1_2 | LINC00395 | antisense | 5.8967 | 0.000 | 0 |
| ENSG00000231310.3_2 | TBL1XR1-AS1 | antisense | 4.0923 | 0.002 | 0 |
| ENSG00000231609.5_2 | AC009501.4 | antisense | 11.8002 | 0.000 | 0 |
| ENSG00000231646.5_2 | FSIP2-AS1 | antisense | 2.6352 | 0.001 | 0 |
| ENSG00000231806.2_2 | PCAT7 | antisense | 11.0574 | 0.000 | 0 |
| ENSG00000232519.2_2 | RP11-29H23.4 | antisense | 3.1566 | 0.002 | 0 |
| ENSG00000234006.1_2 | DDX39B-AS1 | antisense | 3.5816 | 0.003 | 0 |
| ENSG00000234311.1_2 | RP11-432J24.3 | antisense | 4.5736 | 0.001 | 0 |
| ENSG00000234676.1_2 | IFT74-AS1 | antisense | 3.3556 | 0.002 | 0 |
| ENSG00000234949.2_2 | AC104667.3 | antisense | 8.5406 | 0.000 | 0 |
| ENSG00000235029.1_2 | MNX1-AS2 | antisense | 7.3417 | 0.000 | 0 |
| ENSG00000235984.5_2 | GPC5-AS1 | antisense | 26.6618 | 0.000 | 0 |
| ENSG00000236536.1_2 | AC003986.7 | antisense | 7.7715 | 0.000 | 0 |
| ENSG00000237978.5_2 | KCNMB2-AS1 | antisense | 4.3060 | 0.000 | 0 |
| ENSG00000238117.1_2 | AP004372.1 | antisense | 6.0006 | 0.000 | 0 |
| ENSG00000243305.1_2 | RP11-362A9.3 | antisense | 6.1854 | 0.000 | 0 |
| ENSG00000244161.1_2 | FLNB-AS1 | antisense | 8.1513 | 0.000 | 0 |
| ENSG00000244619.2_2 | RP11-315I20.3 | antisense | 6.4611 | 0.000 | 0 |
| ENSG00000244998.1_2 | CTD-3064M3.4 | antisense | 3.7302 | 0.003 | 0 |
| ENSG00000248138.5_2 | RP11-446J8.1 | antisense | 6.2199 | 0.000 | 0 |
| ENSG00000248211.1_2 | TRPC7-AS1 | antisense | 5.9926 | 0.000 | 0 |
| ENSG00000249307.5_2 | LINC01088 | antisense | 3.9132 | 0.000 | 0 |
| ENSG00000249326.1_2 | CTD-2194D22.4 | antisense | 4.3937 | 0.002 | 0 |
| ENSG00000250522.1_2 | AC004066.3 | antisense | 5.0082 | 0.000 | 0 |
| ENSG00000250538.5_2 | RP11-92A5.2 | antisense | 13.8248 | 0.000 | 0 |
| ENSG00000251445.1_2 | RP11-483A20.3 | antisense | 4.8655 | 0.000 | 0 |
| ENSG00000253374.5_3 | RP11-257P3.3 | antisense | 39.1158 | 0.000 | 0 |
| ENSG00000253859.2_3 | RP11-157I4.4 | antisense | 25.9125 | 0.000 | 0 |
| ENSG00000254236.1_2 | KB-1639H6.2 | antisense | 3.5255 | 0.001 | 0 |
| ENSG00000254314.1_2 | RP11-26M5.3 | antisense | 7.2663 | 0.000 | 0 |
| ENSG00000255007.1_2 | CTD-2589M5.4 | antisense | 3.7386 | 0.001 | 0 |
| ENSG00000255301.1_2 | RP11-624G17.3 | antisense | 3.1979 | 0.001 | 0 |
| ENSG00000255348.1_2 | RP11-700F16.3 | antisense | 4.0302 | 0.002 | 0 |
| ENSG00000255523.1_2 | RP11-780O24.2 | antisense | 12.6550 | 0.000 | 0 |
| ENSG00000256947.1_2 | RP11-64D24.2 | antisense | 4.4759 | 0.000 | 0 |
| ENSG00000257434.1_2 | RP11-81K13.1 | antisense | 13.7823 | 0.000 | 0 |
| ENSG00000258162.2_2 | RP11-315E17.1 | antisense | 37.7687 | 0.000 | 0 |
| ENSG00000259342.1_2 | RP11-519G16.5 | antisense | 10.8580 | 0.000 | 0 |
| ENSG00000260228.5_2 | RP11-483P21.2 | antisense | 9.5161 | 0.000 | 0 |
| ENSG00000260788.5_3 | RP11-298D21.1 | antisense | 10.1476 | 0.000 | 0 |
| ENSG00000261441.1_2 | RP11-217B1.2 | antisense | 5.4870 | 0.000 | 0 |
| ENSG00000264116.4_2 | RP11-321M21.3 | antisense | 5.6611 | 0.001 | 0 |
| ENSG00000265678.1_2 | RP11-1376P16.2 | antisense | 4.9356 | 0.001 | 0 |
| ENSG00000265845.2_2 | RP11-20B24.5 | antisense | 7.3287 | 0.000 | 0 |
| ENSG00000267476.1_3 | RP11-126O1.4 | antisense | 7.2147 | 0.000 | 0 |
| ENSG00000267968.1_2 | AC011523.2 | antisense | 7.6059 | 0.000 | 0 |
| ENSG00000269091.5_2 | CTD-2126E3.3 | antisense | 4.3857 | 0.000 | 0 |
| ENSG00000269235.1_2 | ZNF350-AS1 | antisense | 3.9376 | 0.000 | 0 |
| ENSG00000269420.5_2 | PLA2G4C-AS1 | antisense | 10.7720 | 0.000 | 0 |
| ENSG00000271314.1_2 | RP11-435O5.6 | antisense | 4.5142 | 0.000 | 0 |
| ENSG00000277247.1_2 | RP11-785D18.3 | antisense | 3.6184 | 0.002 | 0 |
| ENSG00000279726.1_2 | AC005609.16 | antisense | 6.0826 | 0.000 | 0 |
| ENSG00000280029.3_2 | CH17-140K24.2 | antisense | 4.6338 | 0.002 | 0 |
| ENSG00000182347.10_2 | PDSS1P1 | processed_pseudogene | 6.6507 | 0.000 | 0 |
| ENSG00000213781.3_2 | PSMC1P2 | processed_pseudogene | 6.1490 | 0.001 | 0 |
| ENSG00000215878.3_2 | MARCKSL1P2 | processed_pseudogene | 5.6044 | 0.000 | 0 |
| ENSG00000218549.1_2 | OR4K12P | processed_pseudogene | 13.7342 | 0.000 | 0 |
| ENSG00000226915.1_2 | AC068137.13 | processed_pseudogene | 3.7213 | 0.001 | 0 |
| ENSG00000228767.1_2 | KRT8P18 | processed_pseudogene | 3.4142 | 0.001 | 0 |
| ENSG00000228820.2_2 | RPSAP1 | processed_pseudogene | 5.0900 | 0.000 | 0 |
| ENSG00000231576.1_3 | MTCO2P20 | processed_pseudogene | 8.4181 | 0.000 | 0 |
| ENSG00000232111.2_2 | RP11-126O22.1 | processed_pseudogene | 4.7649 | 0.000 | 0 |
| ENSG00000232176.1_2 | RP11-146N23.1 | processed_pseudogene | 6.7089 | 0.000 | 0 |
| ENSG00000232946.1_2 | RP11-390F4.2 | processed_pseudogene | 4.2588 | 0.000 | 0 |
| ENSG00000234964.4_2 | FABP5P7 | processed_pseudogene | 11.0096 | 0.000 | 0 |
| ENSG00000236044.1_2 | FABP5P2 | processed_pseudogene | 6.5057 | 0.000 | 0 |
| ENSG00000236190.1_2 | RP3-431C21.1 | processed_pseudogene | 4.8595 | 0.001 | 0 |
| ENSG00000237709.1_2 | EEF1A1P28 | processed_pseudogene | 10.5051 | 0.000 | 0 |
| ENSG00000237948.1_3 | MTATP6P20 | processed_pseudogene | 6.7669 | 0.000 | 0 |
| ENSG00000243537.1_2 | CTC-458A3.1 | processed_pseudogene | 8.0025 | 0.000 | 0 |
| ENSG00000249619.1_2 | HMGN1P13 | processed_pseudogene | 3.9434 | 0.001 | 0 |
| ENSG00000251539.1_2 | SNX18P24 | processed_pseudogene | 2.8875 | 0.002 | 0 |
| ENSG00000254567.2_2 | UBTFL9 | processed_pseudogene | 4.1708 | 0.001 | 0 |
| ENSG00000255452.1_2 | RP11-107P7.1 | processed_pseudogene | 4.6221 | 0.001 | 0 |
| ENSG00000256044.1_2 | RP11-324E6.4 | processed_pseudogene | 6.2706 | 0.000 | 0 |
| ENSG00000258440.1_2 | RP11-1033H12.3 | processed_pseudogene | 3.8334 | 0.001 | 0 |
| ENSG00000258527.1_2 | ASB9P1 | processed_pseudogene | 4.6692 | 0.001 | 0 |
| ENSG00000259214.1_2 | RP11-810K23.7 | processed_pseudogene | 11.0380 | 0.000 | 0 |
| ENSG00000259304.1_2 | CTD-2014N11.3 | processed_pseudogene | 4.6478 | 0.000 | 0 |
| ENSG00000270677.1_2 | RP11-294L11.1 | processed_pseudogene | 3.1790 | 0.000 | 0 |
| ENSG00000270727.1_2 | RP11-51E20.1 | processed_pseudogene | 3.9491 | 0.001 | 0 |
| ENSG00000270812.1_2 | CTA-326K9.1 | processed_pseudogene | 8.7152 | 0.000 | 0 |
| ENSG00000271225.1_2 | BNIP3P4 | processed_pseudogene | 3.4624 | 0.001 | 0 |
| ENSG00000278486.1_2 | YRDCP1 | processed_pseudogene | 6.8741 | 0.000 | 0 |
| ENSG00000280012.2_2 | RPL23AP61 | processed_pseudogene | 9.7973 | 0.000 | 0 |
| ENSG00000224676.1 | AP000351.8 | pseudogene | 3.9870 | 0.001 | 0 |
| ENSG00000230553.1 | RP11-481A12.2 | pseudogene | 3.8890 | 0.001 | 0 |
| ENSG00000215367.10_3 | TMED11P | transcribed_unitary_pseudogene | 6.0628 | 0.000 | 0 |
| ENSG00000244227.6_3 | LRRC77P | transcribed_unitary_pseudogene | 9.6601 | 0.000 | 0 |
| ENSG00000180178.10_2 | FAR2P1 | transcribed_unprocessed_pseudogene | 3.1146 | 0.000 | 0 |
| ENSG00000239402.3_2 | CYP4F62P | transcribed_unprocessed_pseudogene | 2.9412 | 0.001 | 0 |
| ENSG00000226950.6_2 | DANCR | processed_transcript | 2.3252 | 0.002 | 0 |
| ENSG00000227646.7_3 | STEAP2-AS1 | processed_transcript | 2.2782 | 0.002 | 0 |
| ENSG00000250546.5_2 | RP11-8L2.1 | processed_transcript | 4.0108 | 0.000 | 0 |
| ENSG00000255545.7_2 | RP11-627G23.1 | processed_transcript | 10.2439 | 0.000 | 0 |
| ENSG00000279472.1_3 | CH17-140K24.4 | processed_transcript | 7.2788 | 0.000 | 0 |
| ENSG00000204666.3_3 | CTD-2126E3.1 | sense_overlapping | 4.7296 | 0.000 | 0 |
| ENSG00000224223.1_3 | VSTM2A-OT1 | sense_overlapping | 16.8868 | 0.000 | 0 |
| ENSG00000238158.6_3 | RP11-420L9.4 | sense_overlapping | 4.3952 | 0.001 | 0 |
| ENSG00000260244.1_2 | RP11-588K22.2 | sense_overlapping | 2.1762 | 0.002 | 0 |

**Supplementary Table 4: MiRNAs experimentally proven to bind and regulate expression of TWIST1, and corresponding REs identified within the lnc-ZNF30-3 transcript**.

| name | TWIST1 | lnc-ZNF30-3 | cancer type | references |
| --- | --- | --- | --- | --- |
| miR-1-3p | 1 | 0 | Prostate cancer | (2,3) |
| miR-106a-5p | 1 | 1 | hepatoma | (4) |
| miR-106b-5p | 1 | 1 | endometrial cancer | (5) |
| miR-129-5p | 2 | 1 | breast cancer | (6,7) |
| miR-145-5p | 1 | 5 | prostate cancer | (8–11) |
| miR-151a-3p | 1 | 3 | breast cancer | (10,12) |
| miR-15a-3p | 1 | 2 | gastric, small cell lung, ovarian cancer | (13–15) |
| miR-16-1-3p | 1 | 1 | gastric, hepatocellular carcinoma | (13,16,17); |
| miR-17-5p | 1 | 1 |  | (18) |
| miR-181a-5p | 1 | 2 | squamous cell carcinoma | (10,19) |
| miR-186-5p | 2 | 5 | ovarian, prostate, breast, gastric cancer, cholangiocarcinoma, glioblastoma, liver cancer, mantle cell lymphoma, Ewing sarcoma | (20–28) |
| miR-194-3p | 1 | 5 | lung adenocarcinoma | (29) |
| miR-20a-5p | 1 | 1 | nasopharyngeal carcinoma | (18) |
| miR-20b-5p | 1 | 1 | nasopharyngeal carcinoma | (18) |
| miR-214-5p | 2 | 4 | Endometrial, pancreatic cancer, cholangiocarcinoma | (30–33) |
| miR-300 | 1 | 4 | osteosarcoma, epithelial cancer | (34–36) |
| miR-320a-5p | 1 | 7 | ovarian cancer | (37) |
| miR-326 | 1 | 5 | hepatocellular carcinoma, endometrial cancer | (38–40) |
| miR-33a-5p | 1 | 1 | non-small cell lung cancer, osteosarcoma, hepatocellular carcinoma | (41–43) |
| miR-33b-5p | 1 | 1 | breast cancer, melanoma | (44,45) |
| miR-361-5p | 1 | 6 | hepatocellular carcinoma, glioma, endometrial cancer | (46–48) |
| miR-363-3p | 1 | 4 | renal cell carcinoma | (49) |
| miR-381-3p | 1 | 4 | gastric cancer | (50) (51) |
| miR-489-3p | 1 | 6 | glioblastoma, colorectal cancer | (52,53) |
| miR-508-3p | 1 | 2 | ovarian cancer | (54) |
| miR-519d-3p | 1 | 1 | gastric cancer | (55) |
| miR-520d-5p | 2 | 5 | nasopharyngeal carcinoma | (18) |
| miR-526b-3p | 1 | 1 | breast cancer | (56) |
| miR-532-5p | 1 | 4 | epithelial ovarian cancer | (57) |
| miR-539-5p | 2 | 8 | glioma | (58,59) |
| miR-543 | 1 | 4 | breast, endometrial, pancreatic, ovarian, gastric cancers | (60,61) |
| miR-580-3p | 1 | 4 | breast cancer, osteosarcoma | (11,62) |
| miR-675-3p | 1 | 2 | hepatocellular carcinoma | (63) |
| miR-876-5p | 1 | 1 | glioblastoma | (64) |
| miR-9-5p | 1 | 6 | cervical cancer | (65) |
| miR-93-3p | 1 | 8 | nasopharyngeal carcinoma | (18) |

**Supplementary Table 5: Number of REs for miRNAs experimentally proven to bind and regulate expression of EMT factors: TWIST1/2, ZEB1/2, SNAIL1/2, and putative REs within lnc-ZNF30-3**

| name | lnc-ZNF30-3 | SNAI1 | SNAI2 | ZEB1 | ZEB2 | TWIST1 | TWIST2 | references |
| --- | --- | --- | --- | --- | --- | --- | --- | --- |
| miR-1-3p | 0 | 0 | 1 | 0 | 0 | 1 | 0 | (2,3) |
| miR-106a-5p | 1 | 0 | 0 | 0 | 0 | 1 | 0 | (4) |
| miR-106b-5p | 1 | 0 | 0 | 0 | 0 | 1 | 0 | (5) |
| miR-1179 | 3 | 0 | 0 | 0 | 5 | 0 | 0 | (66) |
| miR-1199-5p | 3 | 0 | 0 | 1 | 0 | 0 | 0 | (67) |
| miR-122-5p | 2 | 0 | 1 | 0 | 0 | 0 | 0 | (68) |
| miR-124-3p | 3 | 0 | 4 | 0 | 0 | 0 | 0 | (69–75) |
| miR-125b-5p | 3 | 1 | 0 | 0 | 0 | 0 | 0 | (76–82) |
| miR-129-5p | 1 | 0 | 0 | 0 | 7 | 2 | 0 | (6,83,84) |
| miR-130b-5p | 7 | 1 | 0 | 0 | 0 | 0 | 0 | (85) |
| miR-137-3p | 0 | 1 | 0 | 0 | 0 | 0 | 0 | (86,87) |
| miR-137-5p | 2 | 1 | 0 | 0 | 0 | 0 | 0 | (86) |
| miR-138-5p | 1 | 0 | 0 | 0 | 1 | 0 | 1 | (88–94) |
| miR-141-3p | 0 | 0 | 0 | 3 | 4 | 0 | 0 | (95–101) |
| miR-144-3p | 0 | 0 | 0 | 3 | 0 | 0 | 0 | (102) |
| miR-145-5p | 5 | 0 | 0 | 0 | 4 | 1 | 1 | (10,11,103) |
| miR-151a-3p | 1 | 0 | 0 | 0 | 0 | 1 | 0 | (10,12) |
| miR-15a-3p | 2 | 0 | 0 | 0 | 0 | 0 | 0 | (13–15) |
| miR-153-3p | 2 | 1 | 0 | 0 | 0 | 0 | 0 | (104,105) |
| miR-16-1-3p | 1 | 0 | 0 | 0 | 0 | 1 | 0 | (13,16,17); |
| miR-17-5p | 1 | 0 | 0 | 0 | 0 | 1 | 0 | (18) |
| miR-186-5p12/18/22 7:50:00 PM | 5 | 0 | 0 | 0 | 0 | 2 | 0 | (20–28) |
| miR-194-3p | 5 | 0 | 0 | 0 | 0 | 1 | 0 | (29) |
| miR-199a-5p | 4 | 1 | 0 | 0 | 0 | 0 | 0 | (106) |
| miR-199b-3p | 2 | 0 | 0 | 2 | 0 | 0 | 0 | (107) |
| miR-200a-3p | 0 | 0 | 0 | 3 | 4 | 0 | 0 | (108–110,110) |
| miR-200b-3p | 2 | 0 | 2 | 10 | 11 | 0 | 0 | (96,109–113) |
| miR-200c-3p | 2 | 0 | 0 | 10 | 11 | 0 | 0 | (110,112,114) |
| miR-203a-3p | 2 | 2 | 3 | 5 | 9 | 0 | 0 | (110,115–119) |
| miR-204-5p | 9 | 1 | 1 | 0 | 0 | 0 | 0 | (120,121) |
| miR-20a-5p | 1 | 0 | 0 | 0 | 0 | 1 | 0 | (18) |
| miR-20b-5p | 1 | 0 | 0 | 0 | 0 | 1 | 0 | (18) |
| miR-205-3p | 2 | 0 | 0 | 3 | 0 | 0 | 0 | (122) |
| miR-205-5p | 2 | 0 | 0 | 1 | 0 | 0 | 0 | (123–128) |
| miR-211-5p | 9 | 1 | 0 | 1 | 0 | 0 | 0 | (129–131) |
| miR-212-3p | 1 | 0 | 0 | 0 | 2 | 0 | 0 | (132) |
| miR-214-5p | 4 | 0 | 0 | 0 | 0 | 2 | 0 | (33) |
| miR-215-5p | 0 | 0 | 0 | 0 | 1 | 0 | 0 | (133–135) |
| miR-217-3p | 3 | 0 | 0 | 4 | 0 | 0 | 0 | (136,137) |
| miR-217-5p | 1 | 0 | 0 | 2 | 0 | 0 | 0 | (136,138) |
| miR-218-5p | 4 | 0 | 1 | 0 | 1 | 0 | 0 | (139,140) |
| miR-22-3p | 1 | 2 | 0 | 0 | 0 | 0 | 0 | (141,142) |
| miR-22-5p | 3 | 0 | 0 | 0 | 0 | 0 | 1 | (143) |
| miR-300 | 4 | 0 | 0 | 0 | 0 | 1 | 0 | (34–36) |
| miR-30a-3p | 5 | 0 | 0 | 0 | 11 | 0 | 0 | (144,145) |
| miR-30a-5p | 1 | 1 | 2 | 0 | 2 | 0 | 0 | (144–147) |
| miR-30b-5p | 1 | 1 | 0 | 0 | 0 | 0 | 0 | (148–151) |
| miR-30c-5p | 1 | 1 | 0 | 0 | 0 | 0 | 0 | (152–155) |
| miR-30d-5p | 1 | 1 | 0 | 0 | 0 | 0 | 0 | (146,156,157) |
| miR-30e-5p | 1 | 1 | 0 | 0 | 0 | 0 | 0 | (158–160) |
| miR-320a-5p | 7 | 0 | 0 | 0 | 0 | 1 | 0 | (37) |
| miR-326 | 5 | 0 | 0 | 0 | 0 | 1 | 0 | (38,39,161) |
| miR-335-5p | 0 | 0 | 0 | 0 | 2 | 0 | 0 | (162,163) |
| miR-33a-5p | 1 | 0 | 3 | 0 | 0 | 1 | 0 | (41,42,164) |
| miR-33b-5p | 1 | 0 | 0 | 0 | 0 | 1 | 0 | (44,45) |
| miR-34a-5p | 3 | 1 | 0 | 0 | 0 | 0 | 0 | (86,165–170) |
| miR-34b-5p | 2 | 1 | 0 | 0 | 0 | 0 | 0 | (169,170,170) |
| miR-34c-5p | 3 | 1 | 0 | 0 | 0 | 0 | 0 | (169–171) |
| miR-361-5p | 6 | 0 | 0 | 0 | 0 | 1 | 0 | (46–48) |
| miR-363-3p | 4 | 1 | 0 | 0 | 0 | 1 | 0 | (49,172) |
| miR-3662 | 1 | 0 | 0 | 7 | 0 | 0 | 0 | (173) |
| miR-3666 | 0 | 0 | 0 | 3 | 0 | 0 | 0 | (174) |
| miR-381-3p | 4 | 0 | 0 | 0 | 0 | 1 | 0 | (50) (51) |
| miR-409-3p | 2 | 0 | 0 | 5 | 0 | 0 | 0 | (175–178) |
| miR-410-3p | 1 | 2 | 0 | 0 | 0 | 0 | 0 | (179) |
| miR-429 | 2 | 0 | 0 | 10 | 11 | 0 | 0 | (100,109,180–182) |
| miR-455-3p | 3 | 0 | 0 | 1 | 0 | 0 | 0 | (183) |
| miR-486- | 1 | 1 | 0 | 0 | 0 | 0 | 0 | (184) |
| miR-489-3p | 6 | 0 | 0 | 0 | 0 | 1 | 0 | (52,53) |
| miR-491-5p | 2 | 3 | 0 | 0 | 0 | 0 | 0 | (185) |
| miR-502-5p | 4 | 2 | 0 | 0 | 0 | 0 | 0 | (186,187) |
| miR-506-3p | 3 | 0 | 0 | 0 | 1 | 0 | 0 | (188,189) |
| miR-508-3p | 2 | 0 | 0 | 0 | 0 | 1 | 0 | (54) |
| miR-519d-3p | 1 | 0 | 0 | 0 | 0 | 1 | 0 | (55) |
| miR-520d-5p | 4 | 0 | 0 | 0 | 0 | 2 | 0 | (18) |
| miR-526b-3p | 1 | 0 | 0 | 0 | 0 | 1 | 0 | (56) |
| miR-532-5p | 4 | 0 | 0 | 0 | 0 | 1 | 0 | (57) |
| miR-539-5p | 8 | 0 | 0 | 0 | 0 | 1 | 0 | (58,59) |
| miR-543 | 4 | 0 | 0 | 0 | 0 | 1 | 0 | (60,61,190) |
| miR-580-3p | 4 | 0 | 0 | 0 | 0 | 1 | 0 | (11,62) |
| miR-630 | 1 | 0 | 1 | 0 | 0 | 0 | 0 | (191,192) |
| miR-655-3p | 1 | 0 | 0 | 4 | 0 | 0 | 0 |  |
| miR-675-3p | 2 | 0 | 0 | 0 | 0 | 1 | 0 | (63) |
| miR-873-5p | 3 | 0 | 0 | 3 | 0 | 0 | 0 | (193,194) |
| miR-876-5p | 1 | 0 | 0 | 0 | 0 | 1 | 0 | (64) |
| miR-9-5p | 6 | 0 | 0 | 0 | 0 | 1 | 0 | (65) |
| miR-93-3p | 8 | 0 | 0 | 0 | 0 | 1 | 0 | (18) |
| miR-940 | 5 | 3 | 0 | 0 | 0 | 0 | 0 | (195,196) |

**Supplementary References:**

1. Gao J, Ba A, U D, G D, B G, So S, et al. Integrative analysis of complex cancer genomics and clinical profiles using the cBioPortal. Science signaling [Internet]. 2013 Apr 2 [cited 2022 Jun 6];6(269). Available from: https://pubmed.ncbi.nlm.nih.gov/23550210/

2. Chang YS, Chen WY, Yin JJ, Sheppard-Tillman H, Huang J, Liu YN. EGF Receptor Promotes Prostate Cancer Bone Metastasis by Downregulating miR-1 and Activating TWIST1. Cancer Res. 2015 Aug 1;75(15):3077–86.

3. Tominaga E, Yuasa K, Shimazaki S, Hijikata T. MicroRNA-1 targets Slug and endows lung cancer A549 cells with epithelial and anti-tumorigenic properties. Exp Cell Res. 2013 Feb 1;319(3):77–88.

4. Wang R, Li Y, Hou Y, Yang Q, Chen S, Wang X, et al. The PDGF-D/miR-106a/Twist1 pathway orchestrates epithelial-mesenchymal transition in gemcitabine resistance hepatoma cells. Oncotarget. 2015 Mar 30;6(9):7000–10.

5. Dong P, Kaneuchi M, Watari H, Sudo S, Sakuragi N. MicroRNA-106b modulates epithelial-mesenchymal transition by targeting TWIST1 in invasive endometrial cancer cell lines. Mol Carcinog. 2014 May;53(5):349–59.

6. Yao N, Fu Y, Chen L, Liu Z, He J, Zhu Y, et al. Long non-coding RNA NONHSAT101069 promotes epirubicin resistance, migration, and invasion of breast cancer cells through NONHSAT101069/miR-129-5p/Twist1 axis. Oncogene. 2019 Nov;38(47):7216–33.

7. Yu Y, Zhao Y, Sun XH, Ge J, Zhang B, Wang X, et al. Down-regulation of miR-129-5p via the Twist1-Snail feedback loop stimulates the epithelial-mesenchymal transition and is associated with poor prognosis in breast cancer. Oncotarget. 2015 Oct 27;6(33):34423–36.

8. Rajabi F, Liu-Bordes WY, Pinskaya M, Dominika F, Kratassiouk G, Pinna G, et al. CPEB1 orchestrates a fine-tuning of miR-145-5p tumor-suppressive activity on TWIST1 translation in prostate cancer cells. Oncotarget. 2020 Nov 10;11(45):4155–68.

9. Shen X, Jiang H, Chen Z, Lu B, Zhu Y, Mao J, et al. MicroRNA-145 Inhibits Cell Migration and Invasion in Colorectal Cancer by Targeting TWIST. Onco Targets Ther. 2019;12:10799–809.

10. Nairismagi ML, Fuchtbauer A, Labouriau R, Bramsen JB, Fuchtbauer EM. The proto-oncogene TWIST1 is regulated by microRNAs. PLoS One. 2013;8(5):e66070.

11. Nairismagi ML, Vislovukh A, Meng Q, Kratassiouk G, Beldiman C, Petretich M, et al. Translational control of TWIST1 expression in MCF-10A cell lines recapitulating breast cancer progression. Oncogene. 2012 Nov 22;31(47):4960–6.

12. Yeh TC, Huang TT, Yeh TS, Chen YR, Hsu KW, Yin PH, et al. miR-151-3p Targets TWIST1 to Repress Migration of Human Breast Cancer Cells. PLoS One. 2016;11(12):e0168171.

13. Wang T, Hou J, Li Z, Zheng Z, Wei J, Song D, et al. miR-15a-3p and miR-16-1-3p Negatively Regulate Twist1 to Repress Gastric Cancer Cell Invasion and Metastasis. Int J Biol Sci. 2017;13(1):122–34.

14. Wang D, Wu W, Huang W, Wang J, Luo L, Tang D. LncRNA LUADT1 sponges miR-15a-3p to upregulate Twist1 in small cell lung cancer. BMC Pulm Med. 2019 Dec 16;19(1):246.

15. Fan B, Chen LP, Yuan YH, Xiao HN, Lv XS, Xia ZY. MiR-15a-3p suppresses the growth and metastasis of ovarian cancer cell by targeting Twist1. Eur Rev Med Pharmacol Sci. 2019 Mar;23(5):1934–46.

16. Zhang H, Li Z. microRNA-16 Via Twist1 Inhibits EMT Induced by PM2.5 Exposure in Human Hepatocellular Carcinoma. Open Med (Wars). 2019;14:673–82.

17. Feng QQ, Dong ZQ, Zhou Y, Zhang H, Long C. miR-16-1-3p targets TWIST1 to inhibit cell proliferation and invasion in NSCLC. Bratisl Lek Listy. 2018;119(1):60–5.

18. Tsukerman P, Yamin R, Seidel E, Khawaled S, Schmiedel D, Bar-Mag T, et al. MiR-520d-5p directly targets TWIST1 and downregulates the metastamiR miR-10b. Oncotarget. 2014 Dec 15;5(23):12141–50.

19. Liu M, Wang J, Huang H, Hou J, Zhang B, Wang A. miR-181a-Twist1 pathway in the chemoresistance of tongue squamous cell carcinoma. Biochem Biophys Res Commun. 2013 Nov 15;441(2):364–70.

20. Xiong Y, Chen R, Wang L, Wang S, Tu Y, Zhu L, et al. Downregulation of miR‑186 promotes the proliferation and drug resistance of glioblastoma cells by targeting Twist1. Mol Med Rep. 2019 Jun;19(6):5301–8.

21. Zhu X, Shen H, Yin X, Long L, Xie C, Liu Y, et al. miR-186 regulation of Twist1 and ovarian cancer sensitivity to cisplatin. Oncogene. 2016 Jan 21;35(3):323–32.

22. Chang Z, Cui J, Song Y. Long noncoding RNA PVT1 promotes EMT via mediating microRNA-186 targeting of Twist1 in prostate cancer. Gene. 2018 May 15;654:36–42.

23. Zhang M, Shi B, Zhang K. miR-186 Suppresses the Progression of Cholangiocarcinoma Cells Through Inhibition of Twist1. Oncol Res. 2019 Sep 23;27(9):1061–8.

24. Sun WJ, Zhang YN, Xue P. miR-186 inhibits proliferation, migration, and epithelial-mesenchymal transition in breast cancer cells by targeting Twist1. J Cell Biochem. 2019 Jun;120(6):10001–9.

25. Cao C, Sun D, Zhang L, Song L. miR-186 affects the proliferation, invasion and migration of human gastric cancer by inhibition of Twist1. Oncotarget. 2016 Nov 7;7(48):79956–63.

26. Song F, Zhang Y, Pan Z, Zhang Q, Lu X, Huang P. Resveratrol inhibits the migration, invasion and epithelial-mesenchymal transition in liver cancer cells through up- miR-186-5p expression. Zhejiang Da Xue Xue Bao Yi Xue Ban. 2021 Oct 25;50(5):582–90.

27. Mei M, Wang Y, Wang Q, Liu Y, Song W, Zhang M. CircCDYL Serves as a New Biomarker in Mantle Cell Lymphoma and Promotes Cell Proliferation. Cancer Manag Res. 2019;11:10215–21.

28. Mercatelli N, Fortini D, Palombo R, Paronetto MP. Small molecule inhibition of Ewing sarcoma cell growth via targeting the long non coding RNA HULC. Cancer Lett. 2020 Jan 28;469:111–23.

29. Xia W, Mao Q, Chen B, Wang L, Ma W, Liang Y, et al. The TWIST1-centered competing endogenous RNA network promotes proliferation, invasion, and migration of lung adenocarcinoma. Oncogenesis. 2019 Oct 23;8(11):62.

30. Fang YY, Tan MR, Zhou J, Liang L, Liu XY, Zhao K, et al. miR-214-3p inhibits epithelial-to-mesenchymal transition and metastasis of endometrial cancer cells by targeting TWIST1. Onco Targets Ther. 2019;12:9449–58.

31. Liu Y, Meng F, Wang J, Liu M, Yang G, Song R, et al. A Novel Oxoglutarate Dehydrogenase-Like Mediated miR-214/TWIST1 Negative Feedback Loop Inhibits Pancreatic Cancer Growth and Metastasis. Clin Cancer Res. 2019 Sep 1;25(17):5407–21.

32. Li B, Han Q, Zhu Y, Yu Y, Wang J, Jiang X. Down-regulation of miR-214 contributes to intrahepatic cholangiocarcinoma metastasis by targeting Twist. FEBS J. 2012 Jul;279(13):2393–8.

33. Wang Y, Zhang Z. Increased expression of lncRNA SNHG7 promotes the cell viability, migration, and invasion in pre-eclampsia via modulating the miR-214-5p/TWIST1 axis. Hypertens Pregnancy. 2021 Aug;40(3):261–70.

34. Jia J, Yin P, Han G, Xu M, Wang W, Bi W. MicroRNA-300 decreases cell viability, inhibits migration and promotes apoptosis of osteosarcoma cells via downregulation of Twist1. Mol Med Rep. 2017 Sep;16(3):3613–8.

35. Yu J, Xie F, Bao X, Chen W, Xu Q. miR-300 inhibits epithelial to mesenchymal transition and metastasis by targeting Twist in human epithelial cancer. Mol Cancer. 2014 May 24;13:121.

36. Haga CL, Phinney DG. MicroRNAs in the imprinted DLK1-DIO3 region repress the epithelial-to-mesenchymal transition by targeting the TWIST1 protein signaling network. J Biol Chem. 2012 Dec 14;287(51):42695–707.

37. Li C, Duan P, Wang J, Lu X, Cheng J. miR-320 inhibited ovarian cancer oncogenicity via targeting TWIST1 expression. Am J Transl Res. 2017;9(8):3705–13.

38. Wei LQ, Li L, Lu C, Liu J, Chen Y, Wu H. Involvement of H19/miR-326 axis in hepatocellular carcinoma development through modulating TWIST1. J Cell Physiol. 2019 Apr;234(4):5153–62.

39. Liu W, Zhang B, Xu N, Wang MJ, Liu Q. miR-326 regulates EMT and metastasis of endometrial cancer through targeting TWIST1. Eur Rev Med Pharmacol Sci. 2017 Oct;21(17):3787–93.

40. Ghafouri-Fard S, Gholipour M, Hussen BM, Taheri M. The Impact of Long Non-Coding RNAs in the Pathogenesis of Hepatocellular Carcinoma. Front Oncol. 2021;11:649107.

41. Li Y, Chen G, Yan Y, Fan Q. CASC15 promotes epithelial to mesenchymal transition and facilitates malignancy of hepatocellular carcinoma cells by increasing TWIST1 gene expression via miR-33a-5p sponging. Eur J Pharmacol. 2019 Oct 5;860:172589.

42. Pan J, Fang S, Tian H, Zhou C, Zhao X, Tian H, et al. lncRNA JPX/miR-33a-5p/Twist1 axis regulates tumorigenesis and metastasis of lung cancer by activating Wnt/β-catenin signaling. Mol Cancer. 2020 Jan 15;19(1):9.

43. Zhou Y, Huang Z, Wu S, Zang X, Liu M, Shi J. miR-33a is up-regulated in chemoresistant osteosarcoma and promotes osteosarcoma cell resistance to cisplatin by down-regulating TWIST. J Exp Clin Cancer Res. 2014 Jan 27;33:12.

44. Lin Y, Liu AY, Fan C, Zheng H, Li Y, Zhang C, et al. MicroRNA-33b Inhibits Breast Cancer Metastasis by Targeting HMGA2, SALL4 and Twist1. Sci Rep. 2015 Apr 28;5:9995.

45. Zhang P, Huang C, Fu C, Tian Y, Hu Y, Wang B, et al. Cordycepin (3’-deoxyadenosine) suppressed HMGA2, Twist1 and ZEB1-dependent melanoma invasion and metastasis by targeting miR-33b. Oncotarget. 2015;6(12):9834–53.

46. Yin LC, Xiao G, Zhou R, Huang XP, Li NL, Tan CL, et al. MicroRNA-361-5p Inhibits Tumorigenesis and the EMT of HCC by Targeting Twist1. Biomed Res Int. 2020;2020:8891876.

47. Zhang X, Wei C, Li J, Liu J, Qu J. MicroRNA-361-5p inhibits epithelial-to-mesenchymal transition of glioma cells through targeting Twist1. Oncol Rep. 2017 Mar;37(3):1849–56.

48. Ihira K, Dong P, Xiong Y, Watari H, Konno Y, Hanley SJB, et al. EZH2 inhibition suppresses endometrial cancer progression via miR-361/Twist axis. Oncotarget. 2017 Feb 21;8(8):13509–20.

49. Li WZ, Zou Y, Song ZY, Wei ZW, Chen G, Cai QL, et al. Long non-coding RNA SNHG5 affects the invasion and apoptosis of renal cell carcinoma by regulating the miR-363-3p-Twist1 interaction. Am J Transl Res. 2020;12(2):697–707.

50. Yin Y, Li X, Guo Z, Zhou F. MicroRNA‑381 regulates the growth of gastric cancer cell by targeting TWIST1. Mol Med Rep. 2019 Nov;20(5):4376–82.

51. Yz Y, Q M, Q R, Lj X, Qt W, Cp W. miR-381-3p suppresses breast cancer progression by inhibition of epithelial-mesenchymal transition. World journal of surgical oncology [Internet]. 2021 Aug 6 [cited 2022 Jul 2];19(1). Available from: https://pubmed.ncbi.nlm.nih.gov/34362391/

52. Xiao F, Fan W, Huang X, Fang Z, Zheng X. MiR-489 inhibits proliferation and apoptosis of glioblastoma multiforme cells via regulating TWIST1 expression. J BUON. 2020 Dec;25(6):2592–9.

53. Tao Y, Han T, Zhang T, Ma C, Sun C. LncRNA CHRF-induced miR-489 loss promotes metastasis of colorectal cancer via TWIST1/EMT signaling pathway. Oncotarget. 2017 May 30;8(22):36410–22.

54. Zhao L, Wang W, Xu L, Yi T, Zhao X, Wei Y, et al. Integrative network biology analysis identifies miR-508-3p as the determinant for the mesenchymal identity and a strong prognostic biomarker of ovarian cancer. Oncogene. 2019 Mar;38(13):2305–19.

55. Yue H, Tang B, Zhao Y, Niu Y, Yin P, Yang W, et al. MIR-519d suppresses the gastric cancer epithelial-mesenchymal transition via Twist1 and inhibits Wnt/β-catenin signaling pathway. Am J Transl Res. 2017 Aug 15;9(8):3654–64.

56. Liu YQ, Cong YZ, Jiang J, Sheng JZ, Li XH, Zhao M, et al. MiR-526b suppresses cell proliferation, cell invasion and epithelial-mesenchymal transition in breast cancer by targeting Twist1. Eur Rev Med Pharmacol Sci. 2020 Mar;24(6):3113–21.

57. Wei H, Tang QL, Zhang K, Sun JJ, Ding RF. miR-532-5p is a prognostic marker and suppresses cells proliferation and invasion by targeting TWIST1 in epithelial ovarian cancer. Eur Rev Med Pharmacol Sci. 2018 Sep;22(18):5842–50.

58. Yu H, Gao G, Cai J, Song H, Ma Z, Jin X, et al. MiR-539 functions as a tumor suppressor in pancreatic cancer by targeting TWIST1. Exp Mol Pathol. 2019 Jun;108:143–9.

59. Guo J, Cai H, Liu X, Zheng J, Liu Y, Gong W, et al. Long Non-coding RNA LINC00339 Stimulates Glioma Vasculogenic Mimicry Formation by Regulating the miR-539-5p/TWIST1/MMPs Axis. Mol Ther Nucleic Acids. 2018 Mar 2;10:170–86.

60. Yu Q, Zhang Z, He B, Wang H, Shi P, Li Y. MiR-543 functions as tumor suppressor in ovarian cancer by targeting TWIST1. J Biol Regul Homeost Agents. 2020 Feb;34(1):101–10.

61. Bing L, Hong C, Li-Xin S, Wei G. MicroRNA-543 suppresses endometrial cancer oncogenicity via targeting FAK and TWIST1 expression. Arch Gynecol Obstet. 2014 Sep;290(3):533–41.

62. Tang G, Liu L, Xiao Z, Wen S, Chen L, Yang P. CircRAB3IP upregulates twist family BHLH transcription factor (TWIST1) to promote osteosarcoma progression by sponging miR-580-3p. Bioengineered. 2021 Dec;12(1):3385–97.

63. Hernandez JM, Elahi A, Clark CW, Wang J, Humphries LA, Centeno B, et al. miR-675 mediates downregulation of Twist1 and Rb in AFP-secreting hepatocellular carcinoma. Ann Surg Oncol. 2013 Dec;20 Suppl 3:S625-635.

64. Ma B, Xu J, Chen G, Wei D, Gu P, Li L, et al. miR-876-5p exerts tumor suppressor function by targeting TWIST1 and regulating the epithelial-mesenchymal transition in glioblastoma.

65. Babion I, Jaspers A, van Splunter AP, van der Hoorn IAE, Wilting SM, Steenbergen RDM. miR-9-5p Exerts a Dual Role in Cervical Cancer and Targets Transcription Factor TWIST1. Cells. 2019 Dec 26;9(1):E65.

66. Gao HB, Gao FZ, Chen XF. MiRNA-1179 suppresses the metastasis of hepatocellular carcinoma by interacting with ZEB2. Eur Rev Med Pharmacol Sci. 2019 Jun;23(12):5149–57.

67. Diepenbruck M, Tiede S, Saxena M, Ivanek R, Kalathur RKR, Lüönd F, et al. miR-1199-5p and Zeb1 function in a double-negative feedback loop potentially coordinating EMT and tumour metastasis. Nat Commun. 2017 Oct 27;8(1):1168.

68. Jin Y, Wang J, Han J, Luo D, Sun Z. MiR-122 inhibits epithelial-mesenchymal transition in hepatocellular carcinoma by targeting Snail1 and Snail2 and suppressing WNT/β-cadherin signaling pathway. Exp Cell Res. 2017 Nov 15;360(2):210–7.

69. Huang J, Liang Y, Xu M, Xiong J, Wang D, Ding Q. MicroRNA-124 acts as a tumor-suppressive miRNA by inhibiting the expression of Snail2 in osteosarcoma. Oncol Lett. 2018 Apr;15(4):4979–87.

70. Li SL, Gao HL, Lv XK, Hei YR, Li PZ, Zhang JX, et al. MicroRNA-124 inhibits cell invasion and epithelial-mesenchymal transition by directly repressing Snail2 in gastric cancer. Eur Rev Med Pharmacol Sci. 2017 Aug;21(15):3389–96.

71. Liang YJ, Wang QY, Zhou CX, Yin QQ, He M, Yu XT, et al. MiR-124 targets Slug to regulate epithelial-mesenchymal transition and metastasis of breast cancer. Carcinogenesis. 2013 Mar;34(3):713–22.

72. Xia H, Cheung WKC, Ng SS, Jiang X, Jiang S, Sze J, et al. Loss of brain-enriched miR-124 microRNA enhances stem-like traits and invasiveness of glioma cells. J Biol Chem. 2012 Mar 23;287(13):9962–71.

73. Liu YY, Zhang LY, Du WZ. Circular RNA circ-PVT1 contributes to paclitaxel resistance of gastric cancer cells through the regulation of ZEB1 expression by sponging miR-124-3p. Biosci Rep. 2019 Dec 20;39(12):BSR20193045.

74. Z L, X W, W L, L W, L C, H C. miRNA-124 modulates lung carcinoma cell migration and invasion. International journal of clinical pharmacology and therapeutics [Internet]. 2016 Aug [cited 2022 Jul 2];54(8). Available from: https://pubmed.ncbi.nlm.nih.gov/27251409/

75. Hussen BM, Honarmand Tamizkar K, Hidayat HJ, Taheri M, Ghafouri-Fard S. The role of circular RNAs in the development of hepatocellular carcinoma. Pathol Res Pract. 2021 Jul;223:153495.

76. Wang C, Tao W, Ni S, Chen Q. Upregulation of lncRNA snoRNA host gene 6 regulates NUAK family SnF1-like kinase-1 expression by competitively binding microRNA-125b and interacting with Snail1/2 in bladder cancer. J Cell Biochem. 2019 Jan;120(1):357–67.

77. Gradus B, Alon I, Hornstein E. miRNAs control tracheal chondrocyte differentiation. Dev Biol. 2011 Dec 1;360(1):58–65.

78. Goes CP, Vieceli FM, De La Cruz SM, Simões-Costa M, Yan CYI. Scratch2, a Snail Superfamily Member, Is Regulated by miR-125b. Front Cell Dev Biol. 2020;8:769.

79. Liu Z, Liu H, Desai S, Schmitt DC, Zhou M, Khong HT, et al. miR-125b functions as a key mediator for snail-induced stem cell propagation and chemoresistance. J Biol Chem. 2013 Feb 8;288(6):4334–45.

80. Zheng ZN, Huang GZ, Wu QQ, Ye HY, Zeng WS, Lv XZ. NF-κB-mediated lncRNA AC007271.3 promotes carcinogenesis of oral squamous cell carcinoma by regulating miR-125b-2-3p/Slug. Cell Death Dis. 2020 Dec 12;11(12):1055.

81. Ghafouri-Fard S, Khoshbakht T, Hussen BM, Baniahmad A, Taheri M, Samadian M. A review on the role of DANCR in the carcinogenesis. Cancer Cell Int. 2022 May 19;22(1):194.

82. Zhang HM, Fan TT, Li W, Li XX. Expressions and significances of TTF-1 and PTEN in early endometrial cancer. Eur Rev Med Pharmacol Sci. 2017 Jul;21(3 Suppl):20–6.

83. Li X, Li C, Bi H, Bai S, Zhao L, Zhang J, et al. Targeting ZEB2 By microRNA-129 In Non-Small Cell Lung Cancer Suppresses Cell Proliferation, Invasion And Migration Via Regulating Wnt/β-Catenin Signaling Pathway And Epithelial-Mesenchymal Transition. Onco Targets Ther. 2019;12:9165–75.

84. Li X, Wang S, Li Z, Long X, Guo Z, Zhang G, et al. Retracted: NEAT1 induces epithelial-mesenchymal transition and 5-FU resistance through the miR-129/ZEB2 axis in breast cancer. FEBS Lett. 2016 Nov 1;

85. Bai X, Geng J, Zhou Z, Tian J, Li X. MicroRNA-130b improves renal tubulointerstitial fibrosis via repression of Snail-induced epithelial-mesenchymal transition in diabetic nephropathy. Sci Rep. 2016 Feb 3;6:20475.

86. Dong P, Xiong Y, Watari H, Hanley SJB, Konno Y, Ihira K, et al. MiR-137 and miR-34a directly target Snail and inhibit EMT, invasion and sphere-forming ability of ovarian cancer cells. J Exp Clin Cancer Res. 2016 Sep 5;35(1):132.

87. Yuan F, Miao Z, Chen W, Wu F, Wei C, Yong J, et al. Long non-coding RNA PHACTR2-AS1 promotes tongue squamous cell carcinoma metastasis by regulating Snail. J Biochem. 2020 Dec 26;168(6):651–7.

88. Sun DK, Wang JM, Zhang P, Wang YQ. MicroRNA-138 Regulates Metastatic Potential of Bladder Cancer Through ZEB2. Cell Physiol Biochem. 2015;37(6):2366–74.

89. Long L, Huang G, Zhu H, Guo Y, Liu Y, Huo J. Down-regulation of miR-138 promotes colorectal cancer metastasis via directly targeting TWIST2. J Transl Med. 2013 Oct 30;11:275.

90. Zhu D, Gu L, Li Z, Jin W, Lu Q, Ren T. MiR-138-5p suppresses lung adenocarcinoma cell epithelial-mesenchymal transition, proliferation and metastasis by targeting ZEB2. Pathol Res Pract. 2019 May;215(5):861–72.

91. Yan Z, Bi M, Zhang Q, Song Y, Hong S. LncRNA TUG1 promotes the progression of colorectal cancer via the miR-138-5p/ZEB2 axis. Biosci Rep. 2020 Jun 26;40(6):BSR20201025.

92. Gao S, Wang J, Xie J, Zhang T, Dong P. Role of miR-138 in the regulation of larynx carcinoma cell metastases. Tumour Biol. 2015 Oct 24;

93. Liu X, Wang C, Chen Z, Jin Y, Wang Y, Kolokythas A, et al. MicroRNA-138 suppresses epithelial-mesenchymal transition in squamous cell carcinoma cell lines. Biochem J. 2011 Nov 15;440(1):23–31.

94. Jin Z, Guan L, Song Y, Xiang GM, Chen SX, Gao B. MicroRNA-138 regulates chemoresistance in human non-small cell lung cancer via epithelial mesenchymal transition. Eur Rev Med Pharmacol Sci. 2016;20(6):1080–6.

95. Li D, Wang J, Zhang M, Hu X, She J, Qiu X, et al. LncRNA MAGI2-AS3 Is Regulated by BRD4 and Promotes Gastric Cancer Progression via Maintaining ZEB1 Overexpression by Sponging miR-141/200a. Mol Ther Nucleic Acids. 2020 Mar 6;19:109–23.

96. Zheng L, Xu M, Xu J, Wu K, Fang Q, Liang Y, et al. ELF3 promotes epithelial-mesenchymal transition by protecting ZEB1 from miR-141-3p-mediated silencing in hepatocellular carcinoma. Cell Death Dis. 2018 Mar 9;9(3):387.

97. Chen D, Chou FJ, Chen Y, Tian H, Wang Y, You B, et al. Targeting the radiation-induced TR4 nuclear receptor-mediated QKI/circZEB1/miR-141-3p/ZEB1 signaling increases prostate cancer radiosensitivity. Cancer Lett. 2020 Dec 28;495:100–11.

98. Zhou X, Wang Y, Shan B, Han J, Zhu H, Lv Y, et al. The downregulation of miR-200c/141 promotes ZEB1/2 expression and gastric cancer progression. Med Oncol. 2015 Jan;32(1):428.

99. Long ZH, Bai ZG, Song JN, Zheng Z, Li J, Zhang J, et al. miR-141 Inhibits Proliferation and Migration of Colorectal Cancer SW480 Cells. Anticancer Res. 2017 Aug;37(8):4345–52.

100. Nishijima N, Seike M, Soeno C, Chiba M, Miyanaga A, Noro R, et al. miR-200/ZEB axis regulates sensitivity to nintedanib in non-small cell lung cancer cells. Int J Oncol. 2016 Mar;48(3):937–44.

101. Xiong M, Jiang L, Zhou Y, Qiu W, Fang L, Tan R, et al. The miR-200 family regulates TGF-β1-induced renal tubular epithelial to mesenchymal transition through Smad pathway by targeting ZEB1 and ZEB2 expression. Am J Physiol Renal Physiol. 2012 Feb 1;302(3):F369-379.

102. Guan H, Liang W, Xie Z, Li H, Liu J, Liu L, et al. Down-regulation of miR-144 promotes thyroid cancer cell invasion by targeting ZEB1 and ZEB2. Endocrine. 2015 Mar;48(2):566–74.

103. Ren D, Wang M, Guo W, Huang S, Wang Z, Zhao X, et al. Double-negative feedback loop between ZEB2 and miR-145 regulates epithelial-mesenchymal transition and stem cell properties in prostate cancer cells. Cell Tissue Res. 2014 Dec 1;358(3):763–78.

104. Yang G, Li X, Liu J, Huang S, Weng Y, Zhu J, et al. Hsa_circ_0008537 facilitates liver carcinogenesis by upregulating MCL1 and Snail1 expression via miR‑153‑3p. Oncol Rep. 2021 Mar;45(3):1072–82.

105. Li D, Lu Z, Li X, Xu Z, Jiang J, Zheng Z, et al. Human umbilical cord mesenchymal stem cells facilitate the up-regulation of miR-153-3p, whereby attenuating MGO-induced peritoneal fibrosis in rats. J Cell Mol Med. 2018 Jul;22(7):3452–63.

106. Bai J, Jiao WY. Down-Regulation of ZEB1 by miR-199a-3p Overexpression Restrains Tumor Stem-Like Properties and Mitochondrial Function of Non-Small Cell Lung Cancer. Onco Targets Ther. 2020;13:4607–16.

107. Meng L, Li G, Liu X, Jiang J, Zhu M, Sun Y. Decreased Urine miR-199-3p may be a Potential Biomarker for Diabetic Nephropathy via Targeting Zinc Finger E-box-Binding Protein 1. Clin Lab. 2018 Jul 1;64(7):1177–82.

108. Katsuyama E, Yan M, Watanabe KS, Narazaki M, Matsushima S, Yamamura Y, et al. Downregulation of miR-200a-3p, Targeting CtBP2 Complex, Is Involved in the Hypoproduction of IL-2 in Systemic Lupus Erythematosus-Derived T Cells. J Immunol. 2017 Jun 1;198(11):4268–76.

109. Bracken CP, Gregory PA, Kolesnikoff N, Bert AG, Wang J, Shannon MF, et al. A double-negative feedback loop between ZEB1-SIP1 and the microRNA-200 family regulates epithelial-mesenchymal transition. Cancer Res. 2008 Oct 1;68(19):7846–54.

110. Brabletz S, Bajdak K, Meidhof S, Burk U, Niedermann G, Firat E, et al. The ZEB1/miR-200 feedback loop controls Notch signalling in cancer cells. EMBO J. 2011 Feb 16;30(4):770–82.

111. Liu L, Jiang H, Pan H, Zhu X. LncRNA XIST promotes liver cancer progression by acting as a molecular sponge of miR-200b-3p to regulate ZEB1/2 expression. J Int Med Res. 2021 May;49(5):3000605211016211.

112. Gregory PA, Bert AG, Paterson EL, Barry SC, Tsykin A, Farshid G, et al. The miR-200 family and miR-205 regulate epithelial to mesenchymal transition by targeting ZEB1 and SIP1. Nature cell biology. 2008;10:593–601.

113. Kurashige J, Kamohara H, Watanabe M, Hiyoshi Y, Iwatsuki M, Tanaka Y, et al. MicroRNA-200b regulates cell proliferation, invasion, and migration by directly targeting ZEB2 in gastric carcinoma. Ann Surg Oncol. 2012 Jul;19 Suppl 3:S656-664.

114. Jiang Y, Ji X, Liu K, Shi Y, Wang C, Li Y, et al. Exosomal miR-200c-3p negatively regulates the migraion and invasion of lipopolysaccharide (LPS)-stimulated colorectal cancer (CRC). BMC Mol Cell Biol. 2020 Jun 29;21(1):48.

115. An N, Zheng B. MiR-203a-3p Inhibits Pancreatic Cancer Cell Proliferation, EMT, and Apoptosis by Regulating SLUG. Technol Cancer Res Treat. 2020 Dec;19:1533033819898729.

116. Moes M, Le Béchec A, Crespo I, Laurini C, Halavatyi A, Vetter G, et al. A novel network integrating a miRNA-203/SNAI1 feedback loop which regulates epithelial to mesenchymal transition. PLoS One. 2012;7(4):e35440.

117. Liao H, Bai Y, Qiu S, Zheng L, Huang L, Liu T, et al. MiR-203 downregulation is responsible for chemoresistance in human glioblastoma by promoting epithelial-mesenchymal transition via SNAI2. Oncotarget. 2015 Apr 20;6(11):8914–28.

118. Zhang Z, Zhang B, Li W, Fu L, Fu L, Zhu Z, et al. Epigenetic Silencing of miR-203 Upregulates SNAI2 and Contributes to the Invasiveness of Malignant Breast Cancer Cells. Genes Cancer. 2011 Aug;2(8):782–91.

119. Meidhof S, Brabletz S, Lehmann W, Preca BT, Mock K, Ruh M, et al. ZEB1-associated drug resistance in cancer cells is reversed by the class I HDAC inhibitor mocetinostat. EMBO Mol Med. 2015 Jun;7(6):831–47.

120. Liu Z, Long J, Du R, Ge C, Guo K, Xu Y. miR-204 regulates the EMT by targeting snai1 to suppress the invasion and migration of gastric cancer. Tumour Biol. 2016 Jun;37(6):8327–35.

121. Cai S, Liu J, Ma Q, Bao Y, Chen J, Li Y. Coptis inhibited epithelial-mesenchymal transition and fibrogenesis of diabetic nephropathy through lncRNA CLYBL-AS2-miR-204-5p-SNAI1 axis. J Drug Target. 2020 Nov;28(9):939–48.

122. Zhang Z, He X, Xu J, Zhang G, Yang Y, Ma J, et al. Advantages of Restoring miR-205-3p Expression for Better Prognosis of Gastric Cancer via Prevention of Epithelial-mesenchymal Transition. J Gastric Cancer. 2020 Jun;20(2):212–24.

123. Zhan H xiang, Wang Y, Li C, Xu J wei, Zhou B, Zhu J kang, et al. LincRNA-ROR promotes invasion, metastasis and tumor growth in pancreatic cancer through activating ZEB1 pathway. Cancer Letters. 2016 May 1;374(2):261–71.

124. Kalinkova L, Nikolaieva N, Smolkova B, Ciernikova S, Kajo K, Bella V, et al. miR-205-5p Downregulation and ZEB1 Upregulation Characterize the Disseminated Tumor Cells in Patients with Invasive Ductal Breast Cancer. Int J Mol Sci. 2021 Dec 22;23(1):103.

125. Wang D, Cui L, Yang Q, Wang J. Circular RNA circZFPM2 promotes epithelial-mesenchymal transition in endometriosis by regulating miR-205-5p/ZEB1 signalling pathway. Cell Signal. 2021 Nov;87:110145.

126. L L, S L. miR-205-5p inhibits cell migration and invasion in prostatic carcinoma by targeting ZEB1. Oncology letters [Internet]. 2018 Aug [cited 2022 Jul 2];16(2). Available from: https://pubmed.ncbi.nlm.nih.gov/30008858/

127. Wang D, Cui L, Yang Q, Wang J. Corrigendum to “Circular RNA circZFPM2 promotes epithelial-mesenchymal transition in endometriosis by regulating miR-205-5p/ZEB1 signalling pathway” [Cellular Signalling, 87(2021), 110145]. Cell Signal. 2022 Mar;91:110182.

128. Song S, Yu W, Lin S, Zhang M, Wang T, Guo S, et al. LncRNA ADPGK-AS1 promotes pancreatic cancer progression through activating ZEB1-mediated epithelial-mesenchymal transition. Cancer Biol Ther. 2018 Jul 3;19(7):573–83.

129. Wang K, Jin W, Jin P, Fei X, Wang X, Chen X. miR-211-5p Suppresses Metastatic Behavior by Targeting SNAI1 in Renal Cancer. Mol Cancer Res. 2017 Apr;15(4):448–56.

130. Chen G, Huang P, Xie J, Li R. microRNA‑211 suppresses the growth and metastasis of cervical cancer by directly targeting ZEB1. Mol Med Rep. 2018 Jan;17(1):1275–82.

131. Malpeli G, Barbi S, Zupo S, Tosadori G, Scardoni G, Bertolaso A, et al. Identification of microRNAs implicated in the late differentiation stages of normal B cells suggests a central role for miRNA targets ZEB1 and TP53. Oncotarget. 2017 Feb 14;8(7):11809–26.

132. Yang J, Cui R, Liu Y. MicroRNA-212-3p inhibits paclitaxel resistance through regulating epithelial-mesenchymal transition, migration and invasion by targeting ZEB2 in human hepatocellular carcinoma. Oncol Lett. 2020 Oct;20(4):23.

133. Xiong X, He Q, Liu J, Dai R, Zhang H, Cao Z, et al. MicroRNA miR-215-5p Regulates Doxorubicin-induced Cardiomyocyte Injury by Targeting ZEB2. J Cardiovasc Pharmacol. 2021 Oct 1;78(4):622–9.

134. Tu J, Ma L, Zhang M, Zhang J. Long Non-Coding RNA SOX2 Overlapping Transcript Aggravates H9c2 Cell Injury via the miR-215-5p/ZEB2 Axis and Promotes Ischemic Heart Failure in a Rat Model. Tohoku J Exp Med. 2021 Jul;254(3):221–31.

135. Jin J, Wang Y, Zhao L, Zou W, Tan M, He Q. Exosomal miRNA-215-5p Derived from Adipose-Derived Stem Cells Attenuates Epithelial-Mesenchymal Transition of Podocytes by Inhibiting ZEB2. Biomed Res Int. 2020;2020:2685305.

136. Tan YF, Tang L, OuYang WX, Jiang T, Zhang H, Li SJ. β-catenin-coordinated lncRNA MALAT1 up-regulation of ZEB-1 could enhance the telomerase activity in HGF-mediated differentiation of bone marrow mesenchymal stem cells into hepatocytes. Pathol Res Pract. 2019 Mar;215(3):546–54.

137. Bian Y, Gao G, Zhang Q, Qian H, Yu L, Yao N, et al. KCNQ1OT1/miR-217/ZEB1 feedback loop facilitates cell migration and epithelial-mesenchymal transition in colorectal cancer. Cancer Biol Ther. 2019;20(6):886–96.

138. Ding J, Yeh CR, Sun Y, Lin C, Chou J, Ou Z, et al. Estrogen receptor β promotes renal cell carcinoma progression via regulating LncRNA HOTAIR-miR-138/200c/204/217 associated CeRNA network. Oncogene. 2018 Sep;37(37):5037–53.

139. Shi ZM, Wang L, Shen H, Jiang CF, Ge X, Li DM, et al. Downregulation of miR-218 contributes to epithelial–mesenchymal transition and tumor metastasis in lung cancer by targeting Slug/ZEB2 signaling. Oncogene. 2017 May;36(18):2577–88.

140. Wang Y, Liang S, Yu Y, Shi Y, Zheng H. Knockdown of SNHG12 suppresses tumor metastasis and epithelial-mesenchymal transition via the Slug/ZEB2 signaling pathway by targeting miR-218 in NSCLC. Oncol Lett. 2019 Feb;17(2):2356–64.

141. Zhang K, Li XY, Wang ZM, Han ZF, Zhao YH. MiR-22 inhibits lung cancer cell EMT and invasion through targeting Snail. Eur Rev Med Pharmacol Sci. 2017 Aug;21(16):3598–604.

142. Gan L, Lv L, Liao S. Long non‑coding RNA H19 regulates cell growth and metastasis via the miR‑22‑3p/Snail1 axis in gastric cancer. Int J Oncol. 2019 Jun;54(6):2157–68.

143. Han X, Li H, Liu S, Zhao Z. Study on the Potential Mechanism of miR-22-5p in Non-Small-Cell Lung Cancer. Disease Markers. 2022 Sep 6;2022:1–12.

144. Noori J, Sharifi M, Haghjooy Javanmard S. miR-30a Inhibits Melanoma Tumor Metastasis by Targeting the E-cadherin and Zinc Finger E-box Binding Homeobox 2. Adv Biomed Res. 2018;7:143.

145. di Gennaro A, Damiano V, Brisotto G, Armellin M, Perin T, Zucchetto A, et al. A p53/miR-30a/ZEB2 axis controls triple negative breast cancer aggressiveness. Cell Death Differ. 2018 Dec;25(12):2165–80.

146. Kumarswamy R, Mudduluru G, Ceppi P, Muppala S, Kozlowski M, Niklinski J, et al. MicroRNA-30a inhibits epithelial-to-mesenchymal transition by targeting Snai1 and is downregulated in non-small cell lung cancer. Int J Cancer. 2012 May 1;130(9):2044–53.

147. Xiao B, Shi X, Bai J. miR-30a regulates the proliferation and invasion of breast cancer cells by targeting Snail. Oncol Lett. 2019 Jan;17(1):406–13.

148. Wang Y, Liu Y, Zhang L, Bai L, Chen S, Wu H, et al. miR-30b-5p modulate renal epithelial-mesenchymal transition in diabetic nephropathy by directly targeting SNAI1. Biochem Biophys Res Commun. 2021 Jan 8;535:12–8.

149. Xiong Y, Wang Y, Wang L, Huang Y, Xu Y, Xu L, et al. MicroRNA-30b targets Snail to impede epithelial-mesenchymal transition in pancreatic cancer stem cells. J Cancer. 2018;9(12):2147–59.

150. Fan M, Ma X, Wang F, Zhou Z, Zhang J, Zhou D, et al. MicroRNA-30b-5p functions as a metastasis suppressor in colorectal cancer by targeting Rap1b. Cancer Lett. 2020 May 1;477:144–56.

151. Zhang J, Zhang H, Liu J, Tu X, Zang Y, Zhu J, et al. miR-30 inhibits TGF-β1-induced epithelial-to-mesenchymal transition in hepatocyte by targeting Snail1. Biochem Biophys Res Commun. 2012 Jan 20;417(3):1100–5.

152. Ma T, Zhao Y, Lu Q, Lu Y, Liu Z, Xue T, et al. MicroRNA-30c functions as a tumor suppressor via targeting SNAI1 in esophageal squamous cell carcinoma. Biomed Pharmacother. 2018 Feb;98:680–6.

153. Chen X, Chen RX, Wei WS, Li YH, Feng ZH, Tan L, et al. PRMT5 Circular RNA Promotes Metastasis of Urothelial Carcinoma of the Bladder through Sponging miR-30c to Induce Epithelial-Mesenchymal Transition. Clin Cancer Res. 2018 Dec 15;24(24):6319–30.

154. Zheng Z, Guan M, Jia Y, Wang D, Pang R, Lv F, et al. The coordinated roles of miR-26a and miR-30c in regulating TGFβ1-induced epithelial-to-mesenchymal transition in diabetic nephropathy. Sci Rep. 2016 Nov 22;6:37492.

155. Huang J, Yao X, Zhang J, Dong B, Chen Q, Xue W, et al. Hypoxia-induced downregulation of miR-30c promotes epithelial-mesenchymal transition in human renal cell carcinoma. Cancer Sci. 2013 Dec;104(12):1609–17.

156. Ye Z, Zhao L, Li J, Chen W, Li X. miR-30d Blocked Transforming Growth Factor β1-Induced Epithelial-Mesenchymal Transition by Targeting Snail in Ovarian Cancer Cells. Int J Gynecol Cancer. 2015 Nov;25(9):1574–81.

157. Guo Y, Sun P, Guo W, Yin Q, Han J, Sheng S, et al. LncRNA DDX11 antisense RNA 1 promotes EMT process of esophageal squamous cell carcinoma by sponging miR-30d-5p to regulate SNAI1/ZEB2 expression and Wnt/β-catenin pathway. Bioengineered. 2021 Dec;12(2):11425–40.

158. Zhang W, Chang H, Zhang H, Zhang L. MiR-30e Attenuates Isoproterenol-induced Cardiac Fibrosis Through Suppressing Snai1/TGF-β Signaling. J Cardiovasc Pharmacol. 2017 Dec;70(6):362–8.

159. Tanwar VS, Zhang X, Jagannathan L, Jose CC, Cuddapah S. Cadmium exposure upregulates SNAIL through miR-30 repression in human lung epithelial cells. Toxicol Appl Pharmacol. 2019 Jun 15;373:1–9.

160. Liang Z, Tang S, He R, Luo W, Qin S, Jiang H. The effect and mechanism of miR-30e-5p targeting SNAI1 to regulate epithelial-mesenchymal transition on pancreatic cancer. Bioengineered. 2022 Apr;13(4):8013–28.

161. Rao J, Fu J, Meng C, Huang J, Qin X, Zhuang S. LncRNA SNHG3 Promotes Gastric Cancer Cells Proliferation, Migration, and Invasion by Targeting miR-326. J Oncol. 2021;2021:9935410.

162. Kan Q, Su Y, Yang H. MicroRNA-335 is downregulated in papillary thyroid cancer and suppresses cancer cell growth, migration and invasion by directly targeting ZEB2. Oncol Lett. 2017 Dec;14(6):7622–8.

163. Sun Z, Zhang Z, Liu Z, Qiu B, Liu K, Dong G. MicroRNA-335 inhibits invasion and metastasis of colorectal cancer by targeting ZEB2. Med Oncol. 2014 Jun;31(6):982.

164. Zhang ZR, Yang N. MiR-33a-5p inhibits the growth and metastasis of melanoma cells by targeting SNAI2. Neoplasma. 2020 Jul;67(4):813–24.

165. Aida R, Hagiwara K, Okano K, Nakata K, Obata Y, Yamashita T, et al. miR-34a-5p might have an important role for inducing apoptosis by down-regulation of SNAI1 in apigenin-treated lung cancer cells. Mol Biol Rep. 2021 Mar;48(3):2291–7.

166. Li YY, Xu QW, Xu PY, Li WM. MSC-derived exosomal miR-34a/c-5p and miR-29b-3p improve intestinal barrier function by targeting the Snail/Claudins signaling pathway. Life Sci. 2020 Sep 15;257:118017.

167. Jiang X, Ye Z, Jiang Y, Yu W, Fang Q. LncRNA OIP5-AS1 upregulates snail expression by sponging miR-34a to promote ovarian carcinoma cell invasion and migration. Biol Res. 2020 Oct 22;53(1):49.

168. Wang Y, Wu Z, Hu L. The regulatory effects of metformin on the [SNAIL/miR-34]:[ZEB/miR-200] system in the epithelial-mesenchymal transition(EMT) for colorectal cancer(CRC). Eur J Pharmacol. 2018 Sep 5;834:45–53.

169. Kim NH, Kim HS, Li XY, Lee I, Choi HS, Kang SE, et al. A p53/miRNA-34 axis regulates Snail1-dependent cancer cell epithelial-mesenchymal transition. J Cell Biol. 2011 Oct 31;195(3):417–33.

170. Siemens H, Jackstadt R, Hunten S, Kaller M, Menssen A, Gotz U, et al. miR-34 and SNAIL form a double-negative feedback loop to regulate epithelial-mesenchymal transitions. Cell Cycle. 2011 Dec 15;10(24):4256–71.

171. Morata-Tarifa C, Jiménez G, García MA, Entrena JM, Griñán-Lisón C, Aguilera M, et al. Low adherent cancer cell subpopulations are enriched in tumorigenic and metastatic epithelial-to-mesenchymal transition-induced cancer stem-like cells. Sci Rep. 2016 Jan 11;6:18772.

172. Ding J, Xia Y, Yu Z, Wen J, Zhang Z, Zhang Z, et al. Identification of upstream miRNAs of SNAI2 and their influence on the metastasis of gastrointestinal stromal tumors. Cancer Cell Int. 2019;19:289.

173. Zhu L, Liu Z, Dong R, Wang X, Zhang M, Guo X, et al. MicroRNA-3662 targets ZEB1 and attenuates the invasion of the highly aggressive melanoma cell line A375. Cancer Manag Res. 2019;11:5845–56.

174. Li L, Han LY, Yu M, Zhou Q, Xu JC, Li P. Pituitary tumor-transforming gene 1 enhances metastases of cervical cancer cells through miR-3666-regulated ZEB1. Tumour Biol. 2015 Sep 17;

175. Qu R, Chen X, Zhang C. LncRNA ZEB1-AS1/miR-409-3p/ZEB1 feedback loop is involved in the progression of non-small cell lung cancer. Biochem Biophys Res Commun. 2018 Dec 9;507(1–4):450–6.

176. Wu L, Zhang Y, Huang Z, Gu H, Zhou K, Yin X, et al. MiR-409-3p Inhibits Cell Proliferation and Invasion of Osteosarcoma by Targeting Zinc-Finger E-Box-Binding Homeobox-1. Front Pharmacol. 2019;10:137.

177. Ma Z, Li Y, Xu J, Ren Q, Yao J, Tian X. MicroRNA-409-3p regulates cell invasion and metastasis by targeting ZEB1 in breast cancer. IUBMB Life. 2016 May;68(5):394–402.

178. Becker-Greene D, Li H, Perez-Cremades D, Wu W, Bestepe F, Ozdemir D, et al. MiR-409-3p targets a MAP4K3-ZEB1-PLGF signaling axis and controls brown adipose tissue angiogenesis and insulin resistance. Cell Mol Life Sci. 2021 Dec;78(23):7663–79.

179. Zhang YF, Yu Y, Song WZ, Zhang RM, Jin S, Bai JW, et al. miR-410-3p suppresses breast cancer progression by targeting Snail. Oncol Rep. 2016 Jul;36(1):480–6.

180. Wang Y, Dong X, Hu B, Wang XJ, Wang Q, Wang WL. The effects of Micro-429 on inhibition of cervical cancer cells through targeting ZEB1 and CRKL. Biomed Pharmacother. 2016 May;80:311–21.

181. Arunkumar G, Deva Magendhra Rao AK, Manikandan M, Prasanna Srinivasa Rao H, Subbiah S, Ilangovan R, et al. Dysregulation of miR-200 family microRNAs and epithelial-mesenchymal transition markers in oral squamous cell carcinoma. Oncol Lett. 2018 Jan;15(1):649–57.

182. Diaz-Riascos ZV, Ginesta MM, Fabregat J, Serrano T, Busquets J, Buscail L, et al. Expression and Role of MicroRNAs from the miR-200 Family in the Tumor Formation and Metastatic Propensity of Pancreatic Cancer. Mol Ther Nucleic Acids. 2019 Sep 6;17:491–503.

183. Zhan T, Zhu Q, Han Z, Tan J, Liu M, Liu W, et al. miR-455-3p Functions as a Tumor Suppressor by Restraining Wnt/β-Catenin Signaling via TAZ in Pancreatic Cancer. Cancer Manag Res. 2020 Feb 27;12:1483–92.

184. X Z, T Z, K Y, M Z, K W. miR-486-5p suppresses prostate cancer metastasis by targeting Snail and regulating epithelial-mesenchymal transition. OncoTargets and therapy [Internet]. 2016 Nov 8 [cited 2021 Jul 14];9. Available from: https://pubmed.ncbi.nlm.nih.gov/27877055/

185. Yu T, Wang LN, Li W, Zuo QF, Li MM, Zou QM, et al. Downregulation of miR-491-5p promotes gastric cancer metastasis by regulating SNAIL and FGFR4. Cancer Sci. 2018 May;109(5):1393–403.

186. You J, Wang W, Chang HM, Yi Y, Zhao H, Zhu H, et al. The BMP2 Signaling Axis Promotes Invasive Differentiation of Human Trophoblasts. Front Cell Dev Biol. 2021;9:607332.

187. Guo J, Duan H, Li Y, Yang L, Yuan L. A novel circular RNA circ-ZNF652 promotes hepatocellular carcinoma metastasis through inducing snail-mediated epithelial-mesenchymal transition by sponging miR-203/miR-502-5p. Biochem Biophys Res Commun. 2019 Jun 11;513(4):812–9.

188. Wang GJ, Jiao BP, Liu YJ, Li YR, Deng BB. Reactivation of microRNA-506 inhibits gastric carcinoma cell metastasis through ZEB2. Aging (Albany NY). 2019 Mar 28;11(6):1821–31.

189. Fu X, Deng X, Xiao W, Huang B, Yi X, Zou Y. Downregulation of NEAT1 sensitizes gemcitabine-resistant pancreatic cancer cells to gemcitabine through modulation of the miR-506-3p/ZEB2/EMT axis [Internet]. In Review; 2020 Oct [cited 2021 Jul 21]. Available from: https://www.researchsquare.com/article/rs-86053/v1

190. Guowei L, Yanping J. Bioinformatics Analysis of Stem Cell circ-ASB3 Signaling Pathway and Its Affection on Glioma Biological Characteristics. Front Neuroinform. 2022;16:859937.

191. Jin L, Yi J, Gao Y, Han S, He Z, Chen L, et al. MiR-630 inhibits invasion and metastasis in esophageal squamous cell carcinoma. Acta Biochim Biophys Sin (Shanghai). 2016 Sep;48(9):810–9.

192. Sun Y, Cai J, Yu S, Chen S, Li F, Fan C. MiR-630 Inhibits Endothelial-Mesenchymal Transition by Targeting Slug in Traumatic Heterotopic Ossification. Sci Rep. 2016 Mar 4;6:22729.

193. Hu X, Mu Y, Wang J, Zhao Y. LncRNA TDRG1 promotes the metastasis of NSCLC cell through regulating miR-873-5p/ZEB1 axis. J Cell Biochem. 2019 Nov 19;

194. Li G, Xu Y, Wang S, Yan W, Zhao Q, Guo J. MiR-873-5p inhibits cell migration, invasion and epithelial-mesenchymal transition in colorectal cancer via targeting ZEB1. Pathol Res Pract. 2019 Jan;215(1):34–9.

195. Jiang K, Zhao T, Shen M, Zhang F, Duan S, Lei Z, et al. MiR-940 inhibits TGF-β-induced epithelial-mesenchymal transition and cell invasion by targeting Snail in non-small cell lung cancer. J Cancer. 2019;10(12):2735–44.

196. Jiang K, Zhao T, Shen M, Zhang F, Duan S, Lei Z, et al. Correction: MiR-940 inhibits TGF-β-induced epithelial-mesenchymal transition and cell invasion by targeting Snail in non-small cell lung cancer. J Cancer. 2020;11(16):4897–8.
